# Evaluation of dasatinib and ponatinib for the control of CD123 CAR-T cell functionalities

**DOI:** 10.1016/j.omton.2025.201097

**Published:** 2025-11-21

**Authors:** Charles-Frédéric Mantion, Sabeha Biichlé, Xavier Roussel, Gwenaël Rolin, Agathe Lejeune, Tony Labaigt, Elodie Bôle-Richard, Etienne Daguindau, Bernard Royer, Florian Renosi, Olivier Adotevi, Romain Loyon, Maxime Fredon, Francine Garnache-Ottou

**Affiliations:** 1 Université Marie et Louis Pasteur, EFS, INSERM, RIGHT (UMR 1098), 25000 Besançon, France; 2 CHU Besançon, Hematology Department, 25000 Besançon, France; 3 Université Marie et Louis Pasteur, CHU Besançon, CIC INSERM 1431, 25000 Besançon, France; 4 DimaCell Imaging Resource Center, 25000 Besançon, France; 5 FC’ Innov, Bionoveo, 25000 Besançon, France; 6 CHU Besançon, Pharmacology and Toxicology Laboratory, 25000 Besançon, France; 7 CHU Besançon, Hematology and Cellular Immunology Laboratory, 25000 Besançon, France; 8 CARLA Biotherapeutics, 25000 Besançon, France

**Keywords:** MT: Regular Issue, CAR-T cell, CD123, dasatinib, ponatinib, toxicity, endothelial cell, BPDCN, AML

## Abstract

CD123 CAR-T cells (CAR123) represent a promising therapeutic approach for blastic plasmacytoid dendritic cell neoplasm (BPDCN) and CD123^+^ acute myeloid leukemia (AML). However, the pro-inflammatory environment resulting from CAR-T cell activation can induce CD123 upregulation on endothelial cells and potential on-target/off-tumor toxicity. We evaluated the capacity of two tyrosine kinase inhibitors (TKIs), dasatinib and ponatinib, to reversibly inhibit CAR-T cell functions. Using different *in vitro* models of CAR123 co-culture with BPDCN and AML cell lines, we show that both TKIs reduce CAR123 activation phenotype (CD69 and CD25), tumor necrosis factor α (TNF-α) and interferon-γ (IFN-γ) secretion; degranulation (CD107a); and killing of leukemia cells. Moreover, this inhibition was reversible after elimination of the TKIs. However, only dasatinib was effective at clinically relevant concentrations; 50 nM inhibited TNF-α and IFN-γ secretion, with only a slight reduction in cytotoxicity toward leukemia cells and allowed effective control of CAR-T cell cytotoxicity against endothelial cells in relation to the inhibition of cytokine secretion. Thus, dasatinib could be used to minimize potential CAR123 toxicity toward endothelial cells without compromising its anti-leukemic effects. However, a higher dose could be used to completely inhibit CAR-T cell functionality in the event of toxicity.

## Graphical abstract

**Figure:**
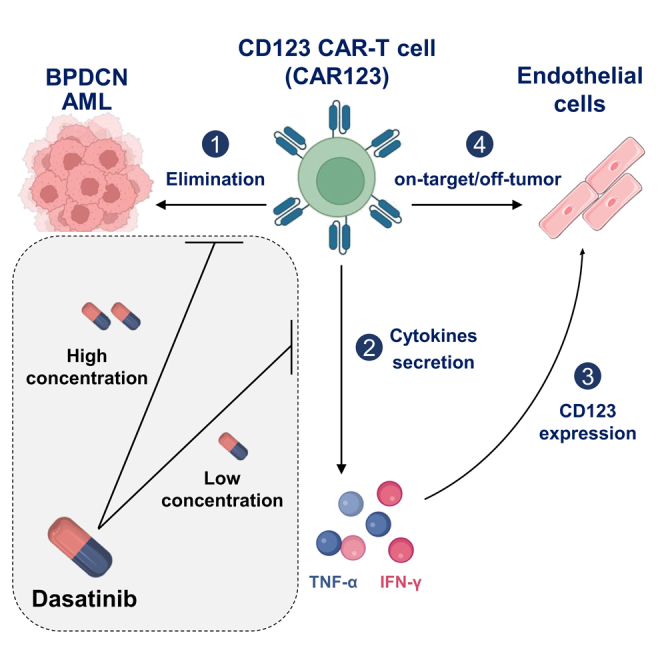

## Teaser

CD123-targeting CAR-T cells may induce an on-target/off-tumor effect on endothelial cells in connection with the cytokine environment linked to CAR-T cell activation. At low concentrations, dasatinib reduces this effect without impacting anti-tumor efficacy, and at high concentrations, it reversibly controls CAR-T cell functionality.

## Introduction

In the last decade, CD19 chimeric antigen receptor (CAR) T cells have demonstrated impressive efficacy in the treatment of B cell leukemia and lymphoma.^1^^,^^2^^,^^3^ However, unwanted toxicity has been observed against healthy cells that also express the target antigen,^4^ commonly referred to as the on-target/off-tumor effect.^5^ CD123 is the α chain of the high-affinity interleukin-3 (IL-3) receptor that is constantly and strongly expressed in blastic plasmacytoid dendritic cell neoplasm (BPDCN),^6^ as well as in the majority of acute myeloid leukemia (AML) cases^7^^,^^8^ and other hemopathies.^9^ Therefore, CD123 represents a highly interesting target antigen for the treatment of various hematological malignancies.^10^ Previously, we selected and developed a CAR-T cell targeting CD123 (CAR123) and evaluated it in different *in vitro* and *in vivo* models of BPDCN and AML.^11^^,^^12^^,^^13^^,^^14^ The CD123 antigen is also strongly expressed by healthy plasmacytoid DCs and basophils and weakly expressed on myeloid progenitors, monocytes, and a small fraction of the most mature hematopoietic stem cells (CD34^+^/CD38^+^).^15^ Importantly, outside hematopoietic cells, CD123 is expressed only by endothelial cells, although at a relatively lower level in their physiological state.^16^ However, the pro-inflammatory environment produced by CAR-T cell activation could increase the expression of CD123.^17^ Interestingly, in human umbilical cord vein endothelial cells (HUVECs), Sun et al. demonstrated that CD123 expression is increased and by interferon-γ (IFN-γ) and tumor necrosis factor α (TNF-α), which increased the cytotoxicity of their CD123 CAR-T cells against HUVECs.^17^ Similarly, in another endothelial cell line, HMEC-1, we observed that IFN-γ and TNF-α induce an increase in CD123 expression.^13^ Thus, it is crucial to implement strategies to manage the activity of CAR-T cells *in vivo* in the event of toxicity, with particular attention on endothelial cells given that the residual toxicity could contribute to the development of capillary leak syndrome (CLS). This syndrome is defined by damage to the capillary barrier, which results in blood loss to surrounding tissues and organs. In CD19 and B cell maturation antigen CAR-T cell therapy, CLS is typically secondary to severe cytokine release syndrome (CRS) rather than being caused by direct toxicity of the CAR-T cells.^18^

To control potential CAR-T cell toxicities, a number of strategies are being explored and evaluated, including the use of “suicide” systems, such as inducible caspase 9,^19^ or the use of therapeutic antibodies to induce the definitive destruction of CAR-T cells.^20^ However, these approaches inducing irreversible CAR-T cell depletion are likely to eliminate the anti-tumor efficacy of the treatment. A preferable approach would be to reversibly inhibit or modulate CAR-T cell functionality, thereby allowing reactivation of CAR-T cells once adverse effects have been controlled. One promising method is the use of tyrosine kinase inhibitors (TKIs), such as dasatinib and ponatinib, that target kinases involved in endogenous T cell receptor (TCR) signaling or CAR-mediated activation.^21^ Dasatinib is already known to reversibly inhibit the functionality of second-generation CAR-T cells *in vitro* and *in vivo*.^22^^,^^23^

The present study evaluated the *in vitro* ability of these two TKIs to reversibly control CAR-T cell activation/cytotoxicity and how this can be used to control the potential endothelial cytotoxicity of CAR123 while maintaining anti-leukemic activity.

## Results

### Effects of TKIs on CAR123 viability and phenotypic activation

**Figure 1:**
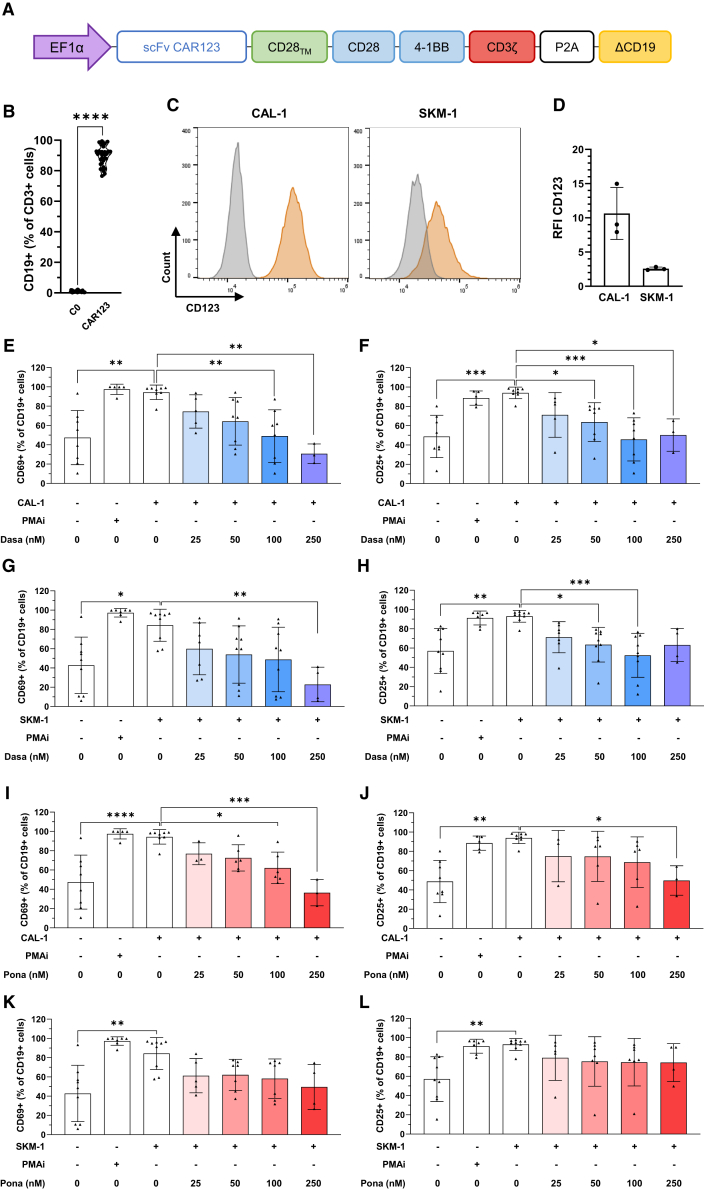
CAR123 design and effect of TKI on its phenotypic activation (A) Schematic representation of CAR123 construct. (B) CAR123 transduction efficiency before use (7–14 days after transduction, *n* = 37 donors). (C) Flow cytometry representation of CAL-1 and SKM-1 CD123 expression. Isotype (gray) and labeling (orange). Data represent a representative experiment (from three independent experiments). (D) CD123 RFI by CAL-1 and SKM-1 (*n* = 3). CAL-1 (E and F) or SKM-1 (G and H) were co-cultured for 18 h at an E:T ratio of 1:1 with CAR123 with or without dasatinib (25–250 nM). CD69 and CD25 expression were evaluated by flow cytometry (*n* ≥ 3 donors). CAL-1 (I and J) or SKM-1 (K and L) were co-cultured for 18 h at an E:T ratio of 1:1 with CAR123 with or without ponatinib (25–250 nM). CD69 and CD25 expression were evaluated by flow cytometry (*n* ≥ 3 donors). Data shown as mean ± SD. ∗*p* < 0.0332, ∗∗*p* < 0.0021, ∗∗∗*p* < 0.0002, and ∗∗∗∗*p* < 0.0001 determined with ordinary one-way ANOVA with Tukey’s multiple comparison test. Values compared to untreated CAR123 in co-culture with CAL-1 or SKM-1.

In the present study, we employed a third-generation CAR-T cell construct targeting CD123 (CAR123) that has been described in detail elsewhere (Figure 1A).^11^^,^^13^ The transduction efficiency was evaluated by flow cytometric analysis using the expression of the selection gene (ΔCD19) contained within the CAR construct on CD3^+^ T cells (CAR123 are CD19^+^/CD3^+^). As a control, non-transduced T cells (C0) from the same donor were produced for each experiment.

The results demonstrated strong transduction on donor primary T cells (89.83% ± 6.29% for CAR123 vs. 0.93% ± 0.51% for C0; Figure 1B). Two CD123^+^ cell lines were used in this study: a BPDCN cell line (CAL-1) with a high CD123 relative fluorescence intensity (RFI) (11.0 ± 3.8) and an AML cell line (SKM-1) with a low CD123 RFI (2.6 ± 0.23; Figures 1C and 1D). Previously, we showed that dasatinib and ponatinib do not reduce the viability of T cells (C0 and CAR123) or target cell lines (CAL-1 and SKM-1; Figures S1A–S1H).

Two common T cell activation markers were evaluated: early marker CD69 and intermediate-late marker CD25. CAR123 was co-cultured with target cell lines in the presence or absence of TKIs to assess the expression of these markers. Following co-culture with CAL-1 cells, we observed a concentration-dependent reduction in both markers in the presence of TKIs, resulting in a significant reduction in CD69 expression by CAR123 using 100 nM dasatinib (Figure 1E) and a reduction in CD25 expression using 50 nM dasatinib (Figure 1F). These findings were corroborated when CAR123 was co-cultured with SKM-1 cells, albeit with slight variation. A significant reduction in CD69 expression was observed at 250 nM dasatinib (Figure 1G), whereas a reduction in CD25 was noted at 50 nM dasatinib (Figure 1H). For treatment with ponatinib, reduced CD69 expression was observed at 100 nM (Figure 1I) and reduced CD25 expression at 250 nM (Figure 1J) with CAL-1 cells. Following co-culture with SKM-1 cells, no significant reduction in activation was observed for CD69 (Figure 1K) or CD25 expression (Figure 1L).

Overall, these data confirm that dasatinib treatment does not induce direct toxicity in T cells or target cell lines, but it does significantly reduce the phenotypic activation profile of activated CAR123 at concentrations as low as 50 nM with CAL-1 and SKM1 cells. In contrast, ponatinib modulates the phenotypic activation of CAR123 only in CAL-1 co-culture and at higher doses than dasatinib.

### Modulation of TNF-α and IFN-γ secretion by TKIs

**Figure 2:**
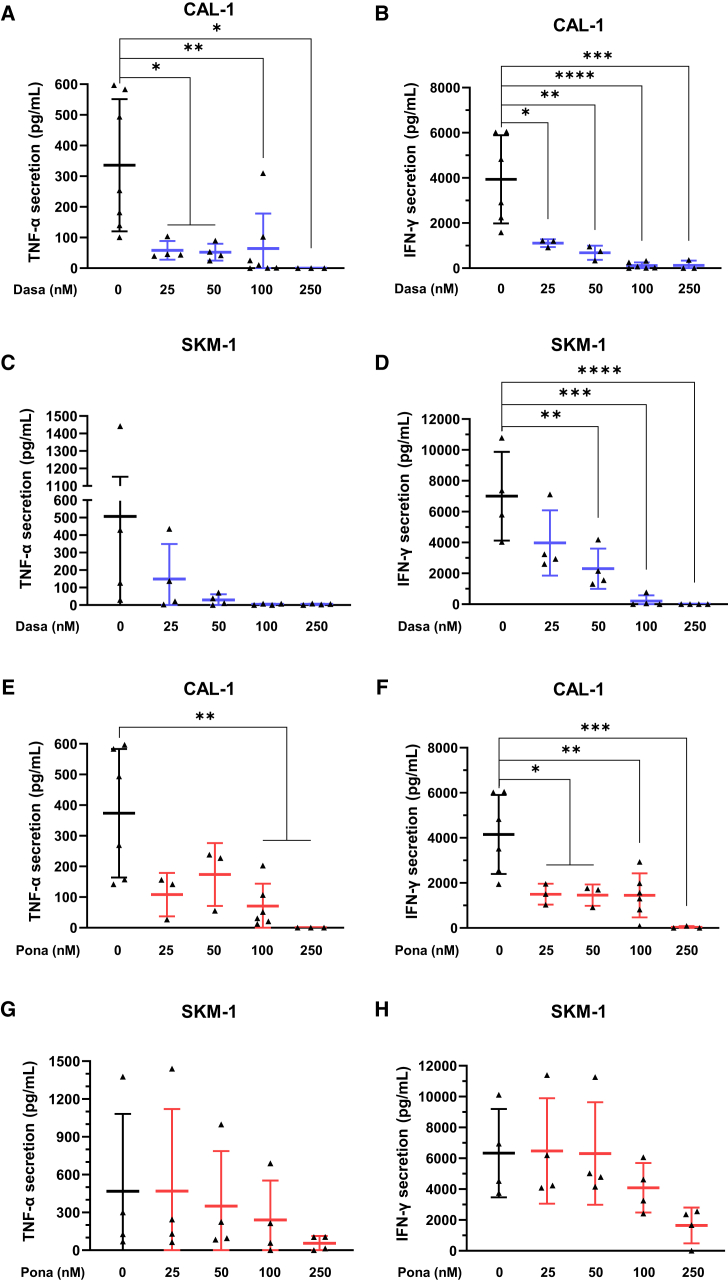
Inhibition of CAR123 TNF-α and IFN-γ secretion by TKI CAL-1 (A and B) or SKM-1 (C and D) were co-cultured for 24 h at an E:T ratio of 1:1 with CAR123 with or without dasatinib (25–250 nM). The secretion of TNF-α and IFN-γ were evaluated by ELISA (*n* ≥ 3 donors). CAL-1 (E and F) or SKM-1 (G and H) were co-cultured for 24 h at an E:T ratio of 1:1 with CAR123 with or without ponatinib (25–250 nM). The secretion of TNF-α and IFN-γ were evaluated by ELISA (*n* ≥ 3 donors). Data shown as mean ± SD. ∗*p* < 0.0332, ∗∗*p* < 0.0021, ∗∗∗*p* < 0.0002, and ∗∗∗∗*p* < 0.0001 determined with ordinary one-way ANOVA with Tukey’s multiple comparison test. Values compared to untreated CAR123 in co-culture with CAL-1 or SKM-1.

TNF-α and IFN-γ are two biomarkers of CAR-T cell activation, and excessive secretion of these cytokines can result in the development of CRS. The ability to control this secretion using TKIs may prove beneficial in reducing these side effects. To this end, we used ELISA to quantify the secretion of IFN-γ and TNF-α by CAR123 following co-culture with target cell lines in the presence of increasing concentrations of TKIs. At concentrations as low as 25 nM, dasatinib significantly reduced TNF-α (Figure 2A) and IFN-γ secretion (Figure 2B) by CAR123 after co-culture with CAL-1 cells. With SKM-1 cells, we observed a tendency toward reduced TNF-α secretion by CAR123 (Figure 2C), and a significant reduction in IFN-γ was observed from a dasatinib concentration of 50 nM (Figure 2D). Ponatinib was able to significantly reduce the secretion of TNF-α from CAR123 against CAL-1 cells at concentrations ≥100 nM (Figure 2E) and to reduce the secretion of IFN-γ at concentrations ≥25 nM (Figure 2F). The reduction was less pronounced with SKM-1 cells, with only a tendency to reduce TNF-α (Figure 2G) and IFN-γ (Figure 2H) at ponatinib concentrations ≥100 nM.

Overall, dasatinib and ponatinib were capable of reducing TNF-α and IFN-γ secretion when CAR123 was activated by its target, particularly at very low doses (25 nM) for dasatinib.

### Modulation of degranulation of CAR123 by TKIs

**Figure 3:**
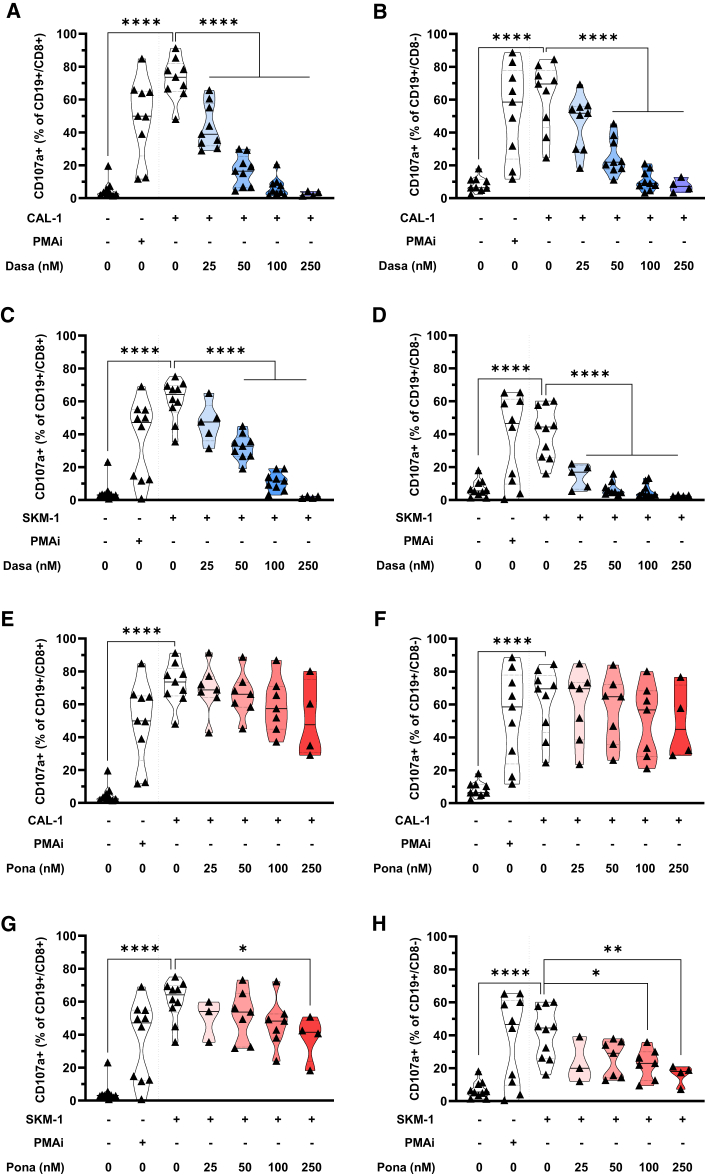
Inhibition of CAR123 degranulation by TKI CAL-1 (A and B) or SKM-1 (C and D) were co-cultured for 5 h at an E:T ratio of 1:1 with CAR123 with or without dasatinib (25–250 nM). CD107a expression among CD8^+^ and CD8^−^ T cells was evaluated by flow cytometry (*n* ≥ 4 donors). CAL-1 (E and F) or SKM-1 (G and H) were co-cultured for 5 h at an E:T ratio of 1:1 with CAR123 with or without ponatinib (25–250 nM). CD107a expression among CD8^+^ or CD8^−^ T cells was evaluated by flow cytometry (*n* ≥ 3 donors). Data shown as median ± quartile. ∗*p* < 0.0332, ∗∗*p* < 0.0021, ∗∗∗*p* < 0.0002, and ∗∗∗∗*p* < 0.0001 determined with ordinary one-way ANOVA with Tukey’s multiple comparison test. Values compared to untreated CAR123 in co-culture with CAL-1 or SKM-1.

The ability to degranulate, as evaluated by CD107a expression, serves as an indicator of CAR-T cell activation and functionality. The expression of CD107a by CD8^+^ and CD8^−^ (indicating CD4^+^ cells) CAR123 was assessed by flow cytometry after co-culture with target cells in the presence of increasing concentrations of TKIs. We observed a significant reduction in CD107a expression at dasatinib concentrations ≥25 nM for CAR123/CD8^+^ (Figure 3A) and ≥50 nM for CAR123/CD8^−^ (Figure 3B) following contact with CAL-1 cells. This reduction was concentration dependent, with a more pronounced effect observed at higher dasatinib concentrations. These findings were validated using SKM-1 cells, showing a significant reduction in CD107a expression in CAR123/CD8^+^ cells at dasatinib concentrations ≥50 nM (Figure 3C) and in CAR123/CD8^−^ cells at dasatinib concentrations ≥25 nM (Figure 3D).

In the case of ponatinib, with CAL-1 cells, ponatinib had no effect on CD107a expression in CAR123/CD8^+^ (Figure 3E) and CAR123/CD8^−^ (Figure 3F). With SKM-1 cells, we observed a significant reduction of CD107a in CAR123/CD8+ (Figure 3G) at a ponatinib concentration of 250 nM and in CAR123/CD8^−^ starting at a ponatinib concentration of 100 nM (Figure 3H).

These results demonstrate that dasatinib can suppress both CD8^+^ and CD8^−^ CAR123 degranulation at concentrations as low as 25 nM, with varying degrees of inhibition depending on the dose used. However, the reduction in degranulation was less pronounced with ponatinib and only significant against SKM-1 cells.

### Reversible control of CAR123 cytotoxicity by TKIs

**Figure 4:**
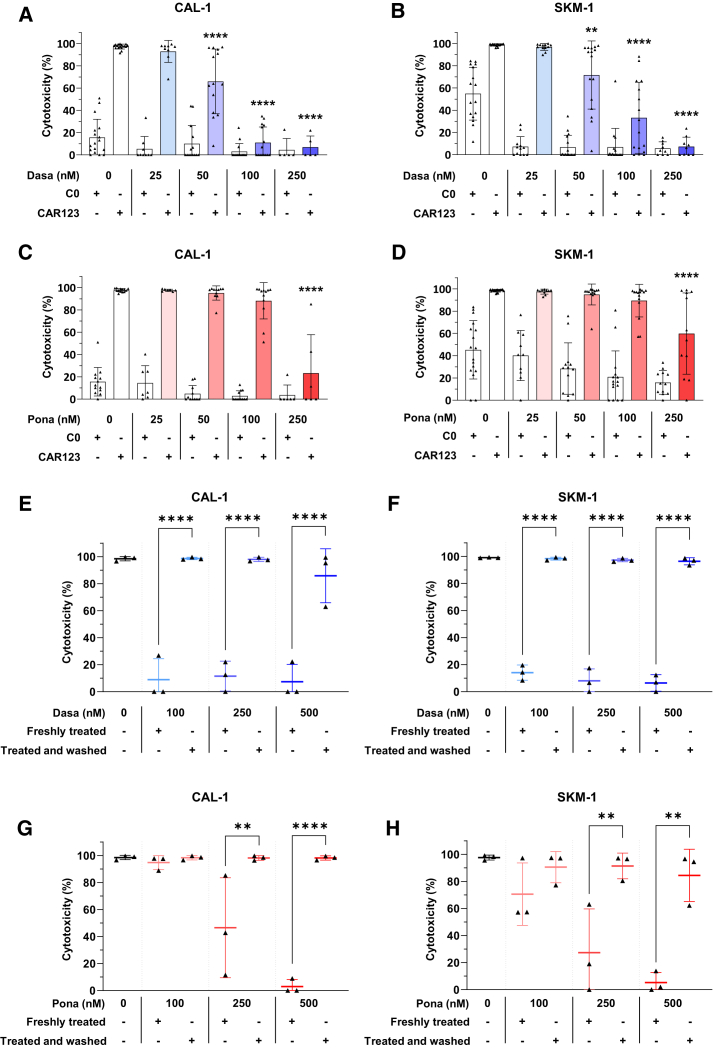
Control of CAR123 cytotoxicity by TKIs CAL-1 (A) or SKM-1 (B) were co-cultured for 24 h at an E:T ratio of 1:1 with CAR123 or C0 with or without dasatinib (25–250 nM). Cytotoxicity of CAR123 is assessed by 7-AAD labeling in flow cytometry (*n* ≥ 5 donors). CAL-1 (C) or SKM-1 (D) were co-cultured for 24 h at an E:T ratio of 1:1 with CAR123 or C0 with or without ponatinib (25–250 nM). Cytotoxicity of CAR123 is assessed by 7-AAD labeling in flow cytometry (*n* ≥ 6 donors). CAL-1 (E) or SKM-1 (F) were co-cultured for 24 h at an E:T ratio of 1:1 with CAR123 or C0 pretreated for 24 h with dasatinib (100–500 nM) before washing or with CAR123 or C0 freshly treated (100–500 nM). Cytotoxicity of CAR123 is assessed by 7-AAD labeling in flow cytometry. Values compared to untreated CAR123 in co-culture with CAL-1 or SKM-1. Data shown as mean ± SD. ∗*p* < 0.0332, ∗∗*p* < 0.0021, ∗∗∗*p* < 0.0002, and ∗∗∗∗*p* < 0.0001 determined with ordinary one-way ANOVA with Tukey’s multiple comparison test. CAL-1 (G) or SKM-1 (H) were co-cultured for 24 h at an E:T ratio of 1:1 with CAR123 or C0 pretreated for 24 h with ponatinib (100–500 nM) before washing or with CAR123 or C0 freshly treated (100–500 nM). Cytotoxicity of CAR123 is assessed by 7-AAD labeling in flow cytometry. Data shown as mean ± SD. ∗*p* < 0.0332, ∗∗*p* < 0.0021, ∗∗∗*p* < 0.0002, and ∗∗∗∗*p* < 0.0001 determined with ordinary one-way ANOVA with Tukey’s multiple comparison test. Values compared to treated CAR123 in co-culture with CAL-1 or SKM-1.

The objective of this study was to evaluate the modulation of CAR123 cytotoxicity by TKIs and to determine whether this inhibition persists after elimination of dasatinib and ponatinib. To this end, co-culture was performed for 24 h with CAR123 or C0, after which cell death was evaluated by flow cytometry. Dasatinib slightly reduced the cytotoxic effect of CAR123 toward CAL-1 (Figure 4A) and SKM-1 cells (Figure 4B) at concentrations ≥50 nM. However, the highest degree of cytotoxicity inhibition was observed with 100 and 250 nM dasatinib for CAL-1 and SKM-1 cells, respectively. In the case of ponatinib, our findings indicate a reduction in the cytotoxic function of CAR123 only at 250 nM for both CAL-1 (Figure 4C) and SKM-1 cells (Figure 4D). Thus, low concentrations of dasatinib (25–50 nM) have no or little effect on the cytotoxicity of CAR123 against leukemic cells. Higher concentrations (≥100 nM) are required to achieve strong inhibition of CAR123 cytotoxicity against leukemic cells. Ponatinib exerted control over activated CAR123 cytotoxicity in both models only at the highest dose (250 nM).

To evaluate the reversibility of this inhibition of cytotoxicity, CAR123 were treated with TKIs and subsequently washed before being co-cultured with CAL-1 or SKM-1 cells as described above. In each experiment, untreated CAR123 (i.e., not treated with TKIs) served as the control group and compared to CAR123 that had recently been treated. The results obtained with untreated CAR123 and freshly treated CAR123 were similar to those observed previously. Washed CAR123, even when treated with a high dose (500 nM) of dasatinib, were able to recover their cytotoxicity against CAL-1 (Figure 4E) and SKM-1 cells (Figure 4F) to a level comparable to that of untreated CAR123. The results observed with ponatinib were similar for CAR123 cultured against CAL-1 (Figure 4G) or SKM-1 cells (Figure 4H).

Taken together, these experiments demonstrate that TKIs induce reversible inhibition of CAR123 cytotoxicity and that CAR123 retains its high cytotoxicity toward leukemia cells even after previous exposure to TKIs. For the remainder of the study, only dasatinib was used due to its functionality at lower concentrations.

### Dasatinib controls the proliferation of CAR123

**Figure 5:**
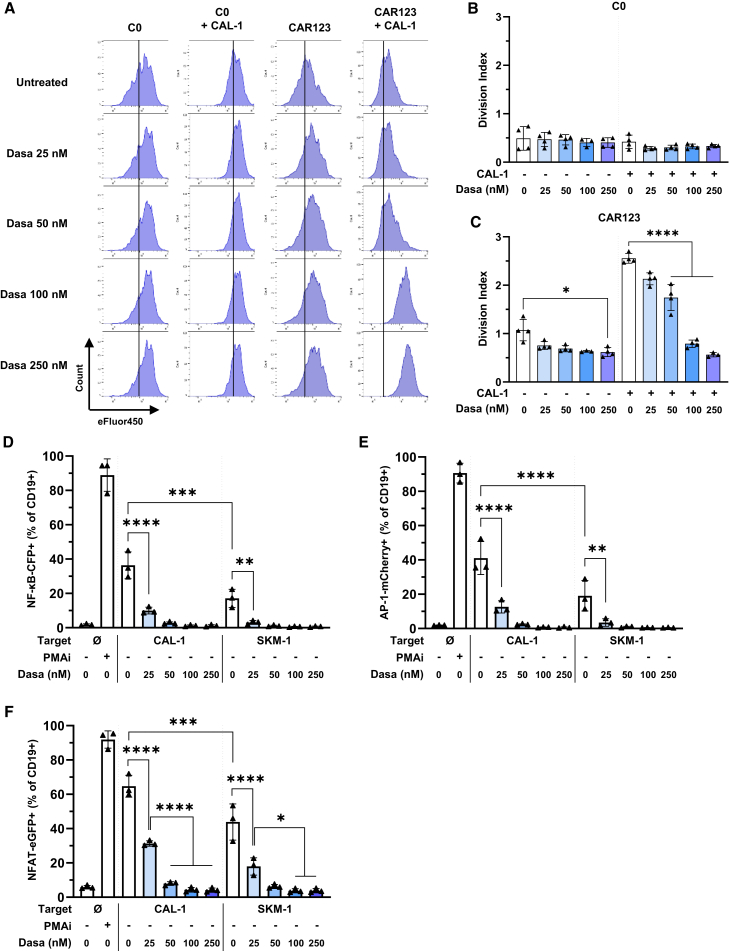
Impact of dasatinib on CAR123 proliferation and activation of transcription factors (A) Representative histograms with eFluor 450 proliferation dye staining of CD3^+^/CD19^−^ cells for C0 or CD3^+^/CD19^+^ cells for CAR. The proliferation assay was performed after 72 h co-culture of C0 or CAR123 with or without CAL-1 at an E:T ratio of 1:1 and dasatinib (25–250 nM). Division index of C0 (B) and CAR123 (C) (*n* ≥ 3 donors). Division index is the mean number of divisions undergone by each cell of the initial generation calculated with FlowJo software. Data shown as mean ± SD. ∗*p* < 0.0332, ∗∗*p* < 0.0021, ∗∗∗*p* < 0.0002, and ∗∗∗∗*p* < 0.0001 determined with ordinary one-way ANOVA with Tukey’s multiple comparison test. Values compared to untreated C0 or CAR123 in co-culture or not. Transcription factors activation assay was performed after 24 h of co-culture of Jurkat TPR CAR123 with or without CAL-1 or SKM-1 at an E:T ratio of 1:1 and dasatinib (25–250 nM). Expression of CFP, mCherry, and EGFP, which is induced upon activation of NF-κB (D), AP-1 (E), and NFAT (F) transcriptional activity, respectively, was assessed by flow cytometry. Data shown as mean ± SD. ∗*p* < 0.0332, ∗∗*p* < 0.0021, ∗∗∗*p* < 0.0002, and ∗∗∗∗*p* < 0.0001 determined with ordinary one-way ANOVA with Tukey’s multiple comparison test. Values compared to Jurkat TPR CAR123 co-cultured with CAL-1 or SKM-1 and treated with 25 nM dasatinib.

We evaluated the ability of dasatinib to control target-induced CAR123 proliferation by determining the division index using flow cytometry and FlowJo software (Figures 5A and S2). As a control, we demonstrated that C0, when cultured alone or in co-culture with CAL-1 cells, did not exhibit any change in the division index whether untreated or treated with increasing concentrations of dasatinib (25–250 nM) (Figures 5A and 5B). In contrast, CAR123 co-cultured with CAL-1 cells had a strong capacity to divide, which was significantly diminished by dasatinib at concentrations ≥50 nM (Figures 5A and 5C). Notably, 50 nM dasatinib was not able to restore CAR123 to their original division index—only concentrations >100 nM were able. Low doses of dasatinib did not reduce the division index of CAR123 when cultured alone; only 250 nM demonstrated a slightly significant effect (Figure 5C).

These results show that the proliferation of CAR123 was not affected by low doses (25 nM) of dasatinib in the presence of its target cells. Intermediate doses (50 nM) induced a significant but modest reduction in proliferation, whereas higher doses (≥100 nM) completely blocked CAR123 proliferation.

### Dasatinib controls activation of NF-κB, AP-1, and NFAT in a Jurkat TPR model

To better characterize the effect of dasatinib on CAR123 downstream signaling, a Jurkat 76 Triple Parameter Reporter (Jurkat TPR) model^24^ was used to assess transcription factor activation. In this model, activation of nuclear factor κB (NF-κB), activator protein 1 (AP-1), or nuclear factor of activated T cells (NFAT) induces expression of the fluorescent proteins cyan fluorescent Protein (CFP), mCherry, or enhanced green fluorescence protein (EGFP), respectively. Jurkat TPR cells were transduced to express CAR123 (Jurkat CAR123) and subsequently co-cultured with CAL-1 or SKM-1 cell lines. Exposure of Jurkat CAR123 cells to target cell lines induced activation of NF-κB (Figure 5D), AP-1 (Figure 5E), and NFAT (Figure 5F). Treatment with 25 nM dasatinib reduced activation of all three transcription factors. Notably, inhibition was less pronounced for NFAT than for NF-κB and AP-1. Higher dasatinib concentrations decreased the activity of all three transcription factors to basal levels. The high inhibition of the three transcription factors beginning at 50 nM suggests that Jurkat CAR123 cells are more sensitive to dasatinib than primary T cells. Comparison of the target cell lines showed that CAL-1 cells induced stronger activation than SKM-1, likely because of the lower CD123 expression of SKM-1 cells.

### Control of on-target/off-tumor effect of CAR123 on endothelial cells

**Figure 6:**
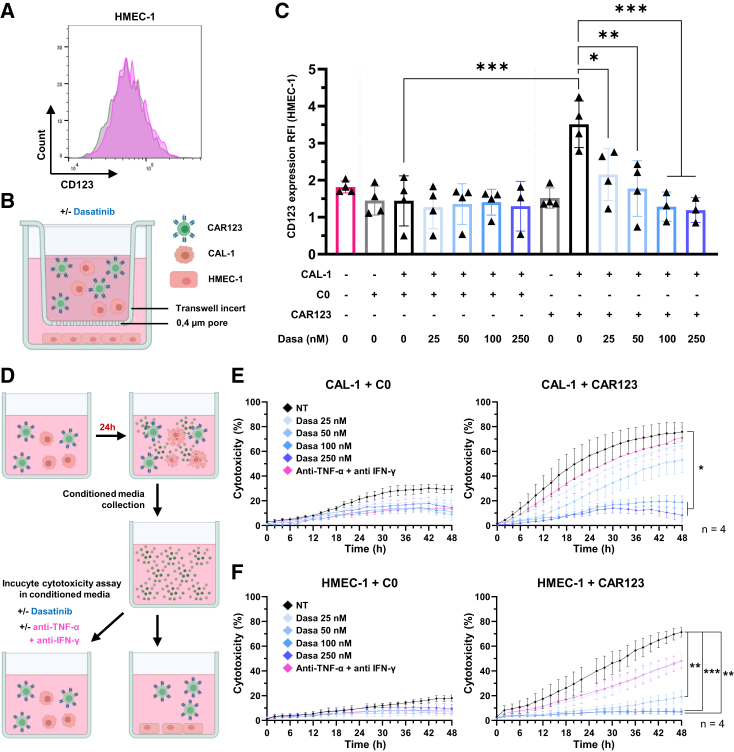
Modulation of the on-target/off-tumor effect of CAR123 on HMEC-1 by dasatinib (A) Flow cytometry representation of HMEC-1 CD123 expression. Isotype (gray) and labeling (pink). Data represent a representative experiment (from four independent experiments). (B) Schematic of the transwell co-culture model. CAR123 or C0 was co-cultured with CAL-1 at an E:T ratio of 1:1 in the upper chamber with or without dasatinib, and HMEC-1s were seeded in the lower chamber. Following 24 h of culture HMEC-1s were digested with trypsin to harvest a single-cell suspension, and CD123 RFI was evaluated by flow cytometry. (C) CD123 RFI of HMEC-1 in the previously described model (*n* ≥ 3 donors). Data shown as mean ± SD. ∗*p* < 0.0332, ∗∗*p* < 0.0021, ∗∗∗*p* < 0.0002, and ∗∗∗∗*p* < 0.0001 determined with ordinary one-way ANOVA with Tukey’s multiple comparison test. Values compared to untreated CAR123 in co-culture with CAL-1. (D) Schematic of the Incucyte S3 model with conditioned media production. CAR123 was co-cultured with CAL-1 for 24 h before collection of the conditioned supernatant. CAL-1 (E) or HMEC-1 (F) were co-cultured for 48 h at an E:T ratio of 1:1 with C0 (left) or CAR123 (right) with or without dasatinib (25–250 nM) or TNF-α/IFN-γ blocking antibodies (5 μg/mL of each) (*n* = 4 donors). CAL-1 or HMEC-1 cell death was followed by the Incucyte S3 system and normalized with viability of target cultured alone. Ordinary two-way ANOVA with Tukey’s multiple comparison test; mean ± SEM. ∗*p* < 0.0332, ∗∗*p* < 0.0021, ∗∗∗*p* < 0.0002, and ∗∗∗∗*p* < 0.0001. Values compared to untreated CAR123.

Finally, we assessed whether the reduction in CAR123 activation by dasatinib could induce a reduction in the on-target/off-tumor effect of CAR123 on endothelial cells. We previously demonstrated that the pro-inflammatory environment (TNF-α and IFN-γ) created by activated CAR123 induced upregulation of CD123 expression on HMEC-1 cells.^13^ We confirmed these data in the tri-culture transwell model (Figures 6A and 6B), as the presence of target cells with CAR123 resulted in increased CD123 expression on HMEC-1 cells in the lower chamber. This increase was counteracted by dasatinib at concentrations ≥25 nM, and 100 nM dasatinib restored basal CD123 expression on endothelial cells (Figure 6C). Dasatinib alone did not affect CD123 expression by HMEC-1 cells (Figure S3A). Similarly, with non-transformed endothelial cells, HUVECs, within the same tri-culture model, the presence of CAL-1 cells with CAR123 induced an increase in CD123 expression on HUVECs. This increase was significantly reduced by treatment with 50 nM dasatinib (Figures S3B and S3C).

The objective of this experiment was to evaluate CAR123 toxicity on HMEC-1 cells and determine whether dasatinib can prevent it. Co-cultures (C0 or CAR123 and HMEC-1) in a pro-inflammatory conditioned medium were treated with dasatinib and cytotoxicity against HMEC-1 cells assessed in real time for 48 h by Incucyte microscopy (Figures 6D and S4). We confirmed that dasatinib alone does not reduce HMEC-1 cell viability (Figures S3D and S3E). In these experiments, we used CAL-1 cells as a positive control for cytotoxicity, as well as anti-IFN-γ and anti-TNF-α blocking antibodies (5 μg/mL each) to highlight the effect of blocking cytokines.

At 48 h, CAR123 was highly cytotoxic against CAL-1 cells (0 nM: 75.8% ± 15.5%), and this cytotoxicity was not reduced by treatment with anti-TNF-α and anti-IFN-γ (71.0% ± 17.4%) or 25 nM dasatinib (64.5% ± 22.4%). The cytotoxicity was slightly reduced at 50 nM dasatinib (53.2% ± 21.2%), and only higher doses reduced it to the level induced by C0 (100 nM: 18.8% ± 10.5%, 250 nM: 8.3% ± 10.8%; Figure 6E and Videos S1 and S2). In this 48-h static model, CAR123 demonstrated cytotoxicity against HMEC-1 cells (0 nM: 71.4% ± 8.1%). Treatment with anti-TNF-α and anti-IFN-γ or 25 nM dasatinib modestly reduced this effect (blocking antibody: 48.1% ± 11.6%, 25 nM: 48.6% ± 10.6%), whereas 50 nM dasatinib was able to significantly reduce cytotoxicity (30.1% ± 7.1%), and higher doses reduced it to the level induced by C0 (100 nM: 6.6% ± 2.0%, 250 nM: 7.2% ± 4.9%; Figure 6F; Videos S3 and S4). To evaluate the potential of dasatinib as a prophylactic measure in this same model, dasatinib was added during production of the conditioned media to mitigate the production of the pro-inflammatory environment. The cytotoxicity toward HMEC-1 cells was comparable (Figures S3F and S3G).

Video S1. Representative Incucyte recording of CAR123 co-cultured with CAL-1 CAL-1 appears red and dead cells appear green. Related to Figure 6E, right.

Video S2. Representative Incucyte recording of CAR123 co-cultured with CAL-1 with 50 nM dasatinib CAL-1 appears red and dead cells appear green. Related to Figure 6E, right.

Video S3. Representative Incucyte recording of CAR123 co-cultured with HMEC-1 HMEC-1 appears red and dead cells appear green. Related to Figure 6F, right.

Video S4. Representative Incucyte recording of CAR123 co-cultured with HMEC-1 with 50 nM dasatinib HMEC-1 appears red and dead cells appear green. Related to Figure 6F, right.

Notably, in these static co-culture models, where cytotoxicity toward HMEC-1 cells was markedly elevated due to the prolonged (48 h) presence of CAR123 on the cell layer, we demonstrated that the lysis of HMEC-1 cells can significantly diminish after treatment with 50 nM dasatinib, whereas the cytotoxicity toward CAL-1 cells remains high at this concentration.

### Modulation of cytotoxicity and TNF-α and IFN-γ secretion by dasatinib

**Figure 7:**
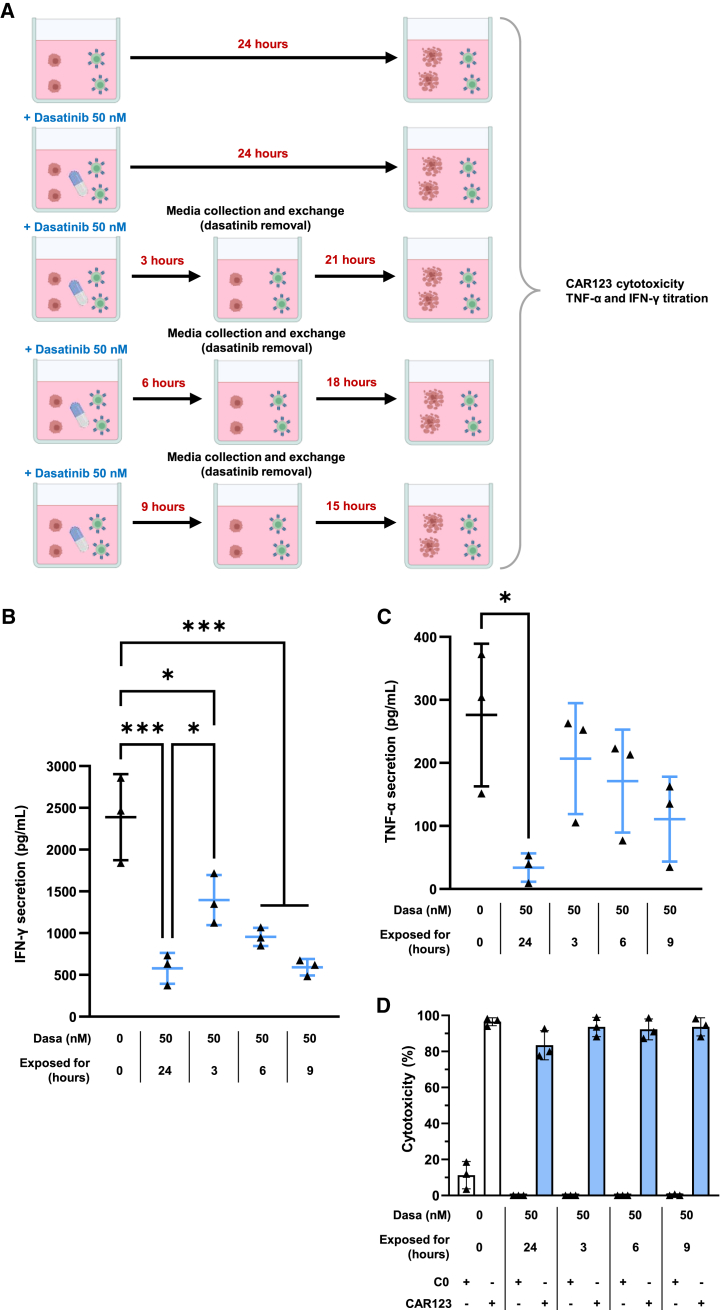
*In vitro* modeling of CAR-T cells’ functionality with dasatinib treatments of varying duration (A) Schematic representation of the co-culture model with varying dasatinib treatment durations. CAR123 was co-cultured with CAL-1 for 24 h. Cells treated with dasatinib were washed after 3, 6, or 9 h of co-culture to remove dasatinib before continuing co-culture. The evaluation of TNF-α and IFN-γ secretion by ELISA (B and C) (*n* = 3 donors) was conducted on collected supernatant when cells were washed and at the end of co-culture. (D) Cytotoxicity of CAR123 on CAL-1 was determined at the end of co-culture by 7-AAD labeling in flow cytometry (*n* = 3 donors). Data shown as mean ± SD. ∗*p* < 0.0332, ∗∗*p* < 0.0021, ∗∗∗*p* < 0.0002, and ∗∗∗∗*p* < 0.0001 determined with ordinary one-way ANOVA with Tukey’s multiple comparison test. Values compared to untreated CAR123 in co-culture with CAL-1 or treated CAR123 in co-culture with CAL-1 without the washing step.

We evaluated whether a different duration of exposure to 50 nM dasatinib (mimicking the dose taken by a patient) could control the overall secretion of TNF-α and IFN-γ by CAR123. We co-cultured CAR123 with CAL-1 cells with or without 50 nM dasatinib. After 3, 6, or 9 h, the culture media was replaced to withdraw dasatinib, and the co-culture continued. The total IFN-γ and TNF-α secretion by CAR123 was evaluated using ELISA. Cytotoxicity toward CAL-1 cells was assessed as a control at the end of the co-culture (Figure 7A). In this model, we demonstrated that a 3-h exposure to 50 nM dasatinib is sufficient to significantly reduce IFN-γ secretion (Figure 7B), with a further reduction observed following 6 and 9 h of exposure. With 9 h of exposure, the IFN-γ level was similar to co-culture treated for the full 24 h. A similar tendency was observed for TNF-α secretion, with reduced secretion following 3, 6, and 9 h of exposure (Figure 7C). In line with prior findings, exposure to 50 nM dasatinib for 3, 6, or 9 h did not result in reduced CAR123 cytotoxicity against CAL-1 cells (Figure 7D).

### Effect of cyclic dasatinib treatments on CAR123 cytotoxicity toward CAL-1 cells in a recursive killing model

**Figure 8:**
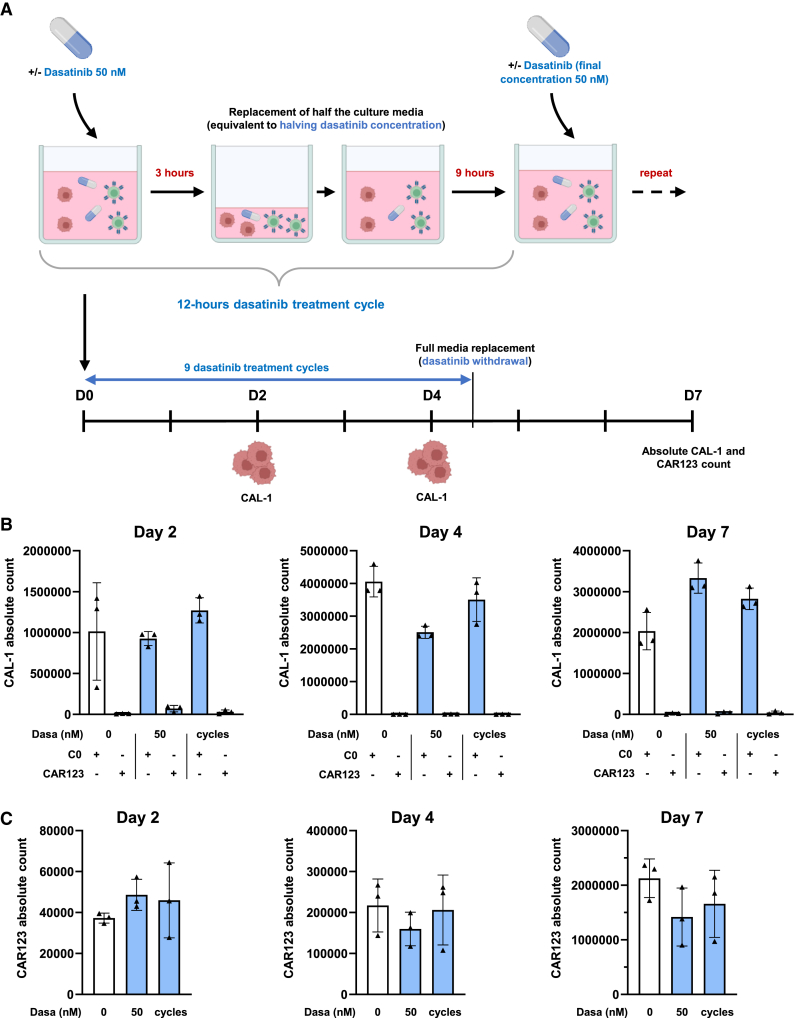
Modulation of CAR123 cytotoxicity on CAL-1 in a recursive killing model by cyclic dasatinib treatments (A) Schematic representation of the recursive killing model and cyclic dasatinib treatments. CAR123 or C0 were co-cultured with CAL-1 at an E:T ratio of 1:5 with or without dasatinib (50 nM, or concentration that alternates between 25 and 50 nM). Absolute number of alive CAL-1 (B) and alive CAR123 (C) was assessed by CD3 and 7-AAD labeling in flow cytometry (*n* = 3 donors) on days 2, 4, and 7 of co-culture. After cytotoxicity evaluation, co-cultures were supplemented with fresh CAL-1 to restore the E:T ratio to 1:5. On day 4, the media was fully replaced, and no dasatinib was added to the co-culture. Data shown as mean ± SD. ∗*p* < 0.0332, ∗∗*p* < 0.0021, ∗∗∗*p* < 0.0002, and ∗∗∗∗*p* < 0.0001 determined with ordinary one-way ANOVA with Tukey’s multiple comparison test. Treated CAR123 values were compared to untreated CAR123.

To evaluate the effect of brief exposure to 50 nM dasatinib on the cytotoxicity of CAR123 toward CAL-1 cells over an extended time period, we employed a recursive killing assay incorporating cycles of dasatinib treatment. CAR123 cells were co-cultured with CAL-1 cells. To emulate cycles of dasatinib treatment, following a 3-h treatment of the co-culture with 50 nM dasatinib, the dasatinib concentration was halved by replacing half of the media. Nine hours after the media replacement, the co-culture was treated again to restore the dasatinib concentration to approximately 50 nM. On days 2 and 4 of the co-culture, the absolute counts of CAL-1 cells and CAR123 were evaluated by flow cytometry and the co-cultures supplemented with fresh CAL-1 cells to restore the effector-to-target (E:T) ratio to 1:5. After nine cycles of dasatinib treatment, on day 4, the media was fully replaced and no more dasatinib added to the co-cultures (Figure 8A). Continuous or cyclic addition of 50 nM dasatinib did not affect the absolute count of CAL-1 cells or CAR123 (Figures 8B and 8C), indicating that multiple exposures to dasatinib did not affect CAR123 cytotoxicity over an extended period of time with rechallenge.

### Effect of dasatinib during CAR123 manufacturing on phenotype and functionality

To determine whether the presence of dasatinib during CAR123 production could serve as an alternative to dasatinib treatment after CAR123 infusion, CAR123 were produced in the presence of 50 nM dasatinib. On day 7, transduction efficiency was evaluated by flow cytometry. CAR123 produced in the presence of dasatinib exhibited lower transduction efficiency than untreated CAR123 (Figure S5A) (untreated: 77% ± 13%; treated: 20% ± 8.2%). CAR123 cells produced in the presence of dasatinib were sorted on day 7. Assessment of transduction efficiency on day 12 in unsorted cells (Figure S5B) indicated that the difference in transduction levels decreased over time. On day 12, the memory phenotype of CAR123 produced with or without dasatinib was evaluated. The presence of dasatinib during CAR123 production resulted in less-advanced lymphocyte differentiation, with a higher proportion of memory T cells (Figure 5C). Following the approach described by Rosselle et al.,^25^ some CAR123 cells produced in the presence of dasatinib were washed 24 h before experimentations to remove the drug. CAR123 produced without dasatinib, CAR123 produced in the presence of dasatinib, and CAR123 produced in the presence of dasatinib followed by washing were co-cultured with or without CAL-1 cells. In parallel, CAR123 produced without dasatinib were extemporaneously treated or not with 50 nM dasatinib immediately before co-culture.

Production of CAR123 in the presence of dasatinib did not significantly affect CD69 or CD25 expression compared with untreated controls (Figures S5D and S5E). Although not statistically significant, CAR123 cells treated extemporaneously with dasatinib displayed a tendency toward lower CD69 expression compared with other conditions (Figure S5D). Regarding programmed cell death protein 1 (PD-1), only CAR123 cells treated extemporaneously with dasatinib exhibited significantly reduced expression, although a similar downward trend was observed in the other conditions (Figure S5F). Collectively, these findings indicate no difference in activation profile between CAR123 produced with dasatinib compared with untreated CAR123.

In terms of degranulation, CAR123 cells produced with dasatinib did not show significantly lower CD107a expression compared with untreated controls. Only CAR123 cells treated extemporaneously with dasatinib demonstrated decreased CD107a expression in both CD3^+^/CD19^+^/CD8^+^ and CD8^−^ populations (Figures S5G and S5H).

Similar patterns were observed in intracellular cytokine expression. In the CD3^+^/CD8^+^ population, only CAR123 cells treated extemporaneously with dasatinib exhibited significantly reduced TNF-α (Figure S6A), IFN-γ (Figure S6B), and IL-2 (Figure S6C) expression. In the CD3^+^/CD8^−^ population, extemporaneous dasatinib treatment significantly decreased TNF-α (Figure S6D) and IL-2 (Figure S6F) expression, with a non-significant trend toward reduced IFN-γ expression (Figure S6E). Conversely, CAR123 cells produced in the presence of dasatinib showed an increase in TNF-α (Figure S6D) and a tendency toward higher IL-2 (Figure S6F) production, potentially reflecting their less-differentiated phenotype. Overall, only extemporaneous dasatinib treatment led to a reduction in cytokine production by CAR-T cells.

Finally, to determine whether dasatinib exposure during manufacturing could eliminate the need for *in vivo* dasatinib administration, a tri-culture transwell model with HUVECs was used to assess CD123 expression. Only extemporaneous dasatinib treatment significantly reduced CD123 expression on HUVECs (Figure S6G).

## Discussion

We previously developed and validated the pre-clinical proof-of-concept of a third-generation CAR-T cell (CD28/4-1BB/CD3ζ) targeting CD123. This CAR-T cell demonstrated high efficiency against BPDCN and AML cells and was chosen among five candidates due to its low cytotoxicity against physiological cells, including hematopoietic progenitors, in both *in vitro* and *in vivo* models of humanized mice.^11^^,^^13^ CD123 is also expressed at low levels on endothelial cells. Therefore, it is crucial to evaluate the CAR123 cytotoxicity toward these cells, especially given that we previously observed a low level of CAR123 activation and cytotoxicity on endothelial cells in various models.^13^ Consequently, we assessed the capacity of activated CAR123 to induce cytotoxicity against endothelial cells and investigated the potential for reversible control of CAR123 cytotoxicity using dasatinib. The literature indicates that other CD123-targeted therapies, such as tagraxofusp (Elzonris, SL-401), may induce endothelial damage^26^ and even fatal CLS (NCT: NCT02113982).^27^^,^^28^ In contrast, no endothelial cell toxicities were reported with an antibody-drug conjugate targeting CD123 (pivekimab sunirine, IMGN632).^29^^,^^30^ Toxicities were manageable with the autologous Mustang Bio CD123 CAR-T cell (MB-102, NCT: NCT02159495),^31^ but one BPDCN patient died on day 9 of CAR-T cell therapy in the Cellectis allogenic CD123 CAR-T cell trial (UCART123, NCT: NCT03203369), probably as a result of CAR-T cell-mediated cardiopulmonary toxicity.

Overall, these data highlight that targeting CD123 can induce toxicity in patients, with the severity of this toxicity depending on the drug being investigated. Therefore, maintaining an equilibrium between functionality and on-target/off-tumor toxicity is essential when evaluating a new drug candidate. As such, the objective of this study was to assess the ability of two TKIs to reversibly control CAR123 functionality.

Dasatinib and ponatinib are broad-spectrum TKIs used for the treatment of BCR::ABL^+^ leukemia.^32^ In humans, the peak steady-state concentration of dasatinib obtained following a standard dosage of 100 mg/day is reported to be 54.6 ng/mL (or 112.2 nM).^33^ In the case of ponatinib, following a standard dosage of 45 mg/day, the steady-state peak plasma concentration is 73.9 ng/mL (or 138.1 nM).^34^ However, at ≤100 nM ponatinib, we observed only minimal effects on CAR123 activation, cytokine secretion, and degranulation. In our model, ponatinib appears to differentially affect CAR123 activation depending on antigen density. In CAL-1 cells with high CD123 expression, ponatinib reduces CD69 expression and cytotoxicity, whereas in SKM-1 cells with lower CD123 expression, its effects are minimal. This observation may reflect the dependence of CAR signaling on the amplitude and duration of phosphorylation, which are modulated by antigen density.^35^ High antigen levels may promote robust kinase recruitment, making the modest inhibition exerted by ponatinib more pronounced, whereas low antigen density, resulting in weaker signaling, is less sensitive to kinase inhibition. Regardless of antigen density, higher ponatinib concentrations (≥250 nM) would be required to achieve inhibition of CAR123 cytotoxicity *in vivo*, which is unlikely to be desirable given the known toxicity of this TKI.^36^ Consequently, the study was continued with only dasatinib; the peak plasma concentration is ∼110 nM,^37^ allowing its use in this concentration range to control CAR123.

Dasatinib presents several interesting properties. The drug did not induce direct toxicity in T cells, CAR123, or HMEC-1 cells. At a starting concentration of 25 nM, we observed a significant reduction in IFN-γ and TNF-α secretion by CAR123 in the presence of CAL-1 cells. However, in the presence of SKM-1 cells, a reduction in cytokine secretion was only observed at a starting concentration of 50 nM. At a starting concentration of 50 nM, dasatinib demonstrated a capacity to regulate target-induced proliferation and activation of CAR123, as evidenced by the expression of CD69 and CD25 and the proliferation assay. At this concentration, a slight reduction in CAR123 cytotoxicity toward CAL-1 or SKM-1 cells was observed. Finally, at concentrations of 100 nM and 250 nM, CAR123 demonstrated a complete loss of functionality, including the absence of cytotoxicity against leukemia cells. The difference in dasatinib concentrations required to inhibit cytokine production and cytotoxicity may be explained by the differential inhibition of AP-1 and NFAT observed in the Jurkat TPR model. In T cells, TCR-induced *TNF* transcription requires simultaneous activation of AP-1 and NFAT.^38^ For *IFNG* transcription, AP-1 activation is essential and depends on NFAT for maximal activity.^39^ Cytotoxic functions, by contrast, are primarily regulated by NFAT in T cells.^40^ In the Jurkat TPR model, treatment with 25 nM dasatinib resulted in a greater reduction of AP-1 than NFAT activity. This finding could account for the decreased TNF-α and IFN-γ secretion observed previously, with minimal impact on cytotoxic function. At higher concentrations, broader inhibition of both AP-1 and NFAT-inducing pathways may suppress both cytokine production and cytotoxic functions.

Notably, the tri-culture transwell model demonstrated that at an initial concentration of 25 nM, dasatinib was capable of significantly reducing CAR123-induced CD123 upregulation in HMEC-1 cells. This reduction is consistent with the observed decrease in IFN-γ and TNF-α secretion. In terms of functionality, the Incucyte co-culture model revealed that CAR123 cytotoxicity was significantly higher toward the adherent HMEC-1 cells than what has been observed in other models. Nevertheless, 25 nM dasatinib moderately reduced cytotoxicity toward HMEC-1 cells but did not reduce the cytotoxicity of CAR123 toward CAL-1 cells. The same reduction in cytotoxicity was observed when anti-IFN-γ and anti-TNF-α were added, indicating that the effect of 25 nM dasatinib is probably due to the inhibition of IFN-γ and TNF-α secretion by CAR123. In this model, the optimal functionality and toxicity balance was achieved with 50 nM dasatinib, with which CAL-1 cells were still eliminated and HMEC-1 cells were relatively spared.

The data presented here suggest that dasatinib may be an effective agent for reducing the secretion of IFN-γ and TNF-α by activated CAR123, thereby reducing CD123 upregulation on endothelial cells and minimizing the on-target/off-tumor effect of CAR123. Furthermore, IFN-γ and TNF-α are known to induce CRS and immune effector cell-associated neurotoxicity syndrome (ICANS) via the activation of other immune cells. Consequently, dasatinib treatment could also be employed to control these side effects as has been reported with a dose of 100 mg/day from day 7–13 following tisagenlecleucel injection^41^ or from day 2–13 post-infusion (dose undisclosed) of GD2 CAR-T cells.^42^

By focusing on the concentration-dependent effects of dasatinib, we demonstrated that 25 nM dasatinib has a slight inhibitory effect on cytokines secreted by activated CAR123 and the cytotoxicity of CAR123 against HMEC-1 cells. At 50 nM, dasatinib exerts a markedly greater inhibitory effect on cytokine secretion and cytotoxicity against HMEC-1 cells while maintaining high cytotoxicity against leukemia cells. At higher doses (≥100 nM), dasatinib fully inhibits CAR123 activity in both endothelial cells and leukemia cells. However, in real life, the dasatinib concentration would vary with pharmacokinetics. The administration of 70 mg dasatinib twice daily (i.e., a dose typically utilized for the treatment of CML, 140 mg/day) has been demonstrated to induce a plasma concentration >50 nM for approximately 3 h.^37^ We developed *in vitro* models to replicate brief dasatinib exposures, confirming the modulation of IFN-γ and TNF-α secretion and low inhibition of cytotoxicity against CAL-1 cells. The data presented here indicate that *in vitro*, a 3-h exposure to dasatinib at a concentration of 50 nM is sufficient to reduce IFN-γ and TNF-α secretion by CAR123. We then confirmed that multiple cycles of dasatinib and rechallenge did not affect the long-term cytotoxicity of CAR123 toward CAL-1 cells. Collectively, these findings suggest that dasatinib may be utilized at clinically relevant concentrations to mitigate the on-target/off-tumor effect toward endothelial cells. This could be exploited as a prophylactic measure at the start of treatment to limit the initial cytokine secretion associated with CAR123 activation, thus avoiding the potential on target-tumor problem without compromising the direct cytotoxicity against leukemia cells. This would also limit the CRS and ICANS associated with the initial cytokine storm.

Our recursive killing assay with cyclic dasatinib treatment suggests that dasatinib exposure will not hinder the long-term cytotoxicity against leukemia cells. Conversely, higher doses of dasatinib can completely inhibit CAR123 functionality for a given period of time. In both cases, after a brief period of reduced CAR123 activation, dasatinib can be discontinued, allowing CAR123 to regain its efficacy against leukemia cells.

Long-term treatment with dasatinib is associated with adverse effects,^43^ particularly pleural effusion. However, dasatinib-associated toxicities are generally more manageable than the severe toxicities related to CAR-T cells, especially when considering its use in short-term treatment regimens. The short-term use of dasatinib to manage CAR-T cell-related toxicities seems to offer a favorable benefit-risk profile. Thus, our work highlights that dasatinib enables personalized control of CAR-T cells, with low doses used to limit initial activation and associated toxicities or higher doses to address proven toxicity. In both scenarios, these effects will resolve upon discontinuation of the drug, allowing CAR-T cells to resume their anti-leukemic activity.

This study has certain limitations related to the study models, however. When assessing CAR123 activation markers or functionality, the results depend on both incubation time and the degree of antigenic stimulation.

Indeed, distinct activation thresholds and temporal delays between stimulation and the appearance of activation markers or effector responses are involved, depending on the phenomenon observed. For example, observed reductions with dasatinib may instead reflect a dasatinib-induced delay in the onset of activation marker expression. Therefore, varying levels of inhibition might have been observed using different incubation times, other E:T ratios, or different target cell types.

Another limitation concerns the extrapolation of *in vitro* findings to *in vivo* conditions. CLS is a multifactorial process regulated by cytokine networks and cellular interactions, which complicates the translation of *in vitro* results to *in vivo* settings. Upregulation of CD123 expression on endothelial cells may be responsible for the on-target/off-tumor effect of CAR123. However, few available *in vitro* or *in vivo* models are relevant for evaluating this process. For instance, murine CD123 shares only 30% sequence homology with human CD123, limiting the use of murine models. Moreover, CAR-T cells tested in immunodeficient mouse strains (e.g., NSG, NXG) lack the myeloid and lymphoid compartments critical for CRS and CLS development. Although humanized models partially restore immune diversity, they exhibit aberrant CD123 expression, impairing accurate assessment of on-target/off-tumor effects.^13^ Consequently, *in vitro* observations cannot be reliably validated *in vivo*, highlighting the current gap in preclinical modeling. Vigilant clinical monitoring following CAR123 infusion is therefore essential to mitigate potential toxicity risks.

## Materials and methods

### Cell lines and culture

Two leukemia cell lines were used for this study: CAL-1 (BPDCN cell line), provided by Dr. Maeda, University of Nagasaki, Japan (RRID: CVCL_5G46) and SKM-1 (AML M5), provided by Deutsche Sammlung von Mikroorganismen und Zelkulturen, (catalog no. ACC 547, RRID: CVCL_0098). One endothelial cell line was used, HMEC-1, provided by American Type Culture Collection (ATCC, Manassas, VA) (catalog no. CRL-3246, RRID: CVCL_0307). Jurkat TPR were provided by Dr. Sylvain Simon, Fred Hutchinson Cancer Research Center (Seattle, WA). Non-transformed HUVECs were provided from Thermo Fisher Scientific (Waltham, MA, catalog no. C0035C, lot no. 1739969). To produce viral vector particles, we used HEK293T, provided from ATCC (catalog no. CRL-11268, RRID: CVCL_1926). Leukemia cell lines and Jurkat TPR were cultivated in RPMI 1640 medium (catalog no. 72400-021, Gibco, Carlsbad, CA) supplemented with 10% fetal bovine serum (FBS) (catalog no. 10270-106, Gibco) and 1% antibiotics (penicillin 10,000 UI/streptomycin 10,000 μg; catalog no. CABPES01-0U, Eurobio, Les Ulis, France). HMEC-1 was cultivated in DMEM (catalog no. L0103-500, Dominique Dutscher, Brussels, Belgium) supplemented by 10% FBS, 1% antibiotics, 10 ng/mL epidermal growth factor (Bio-Techne, Minneapolis, MN, catalog no. 236-EG) and 1 μg/mL hydrocortisone (catalog no. H6909, Sigma-Aldrich, St. Louis, MO). HUVECs were cultivated in endothelial cell growth medium MV 2 (catalog no. C-22022, PromoCell, Heidelberg, Germany). HEK293T was cultivated in DMEM supplemented by 10% FBS and 1% antibiotics. Adherent HMEC-1, HUVEC, and HEK293T cells were taken from the flask wall using trypsin-EDTA 0.5% (catalog no. CEZTDA00-0U, Eurobio) when the confluence of the latter reached 80%.

All cell lines discussed in this work were from passages 8–12, with a maximum culture period of 21 days, except for Jurkat TPR. Mycoplasma was tested on all cell types at the end of culture using the VenorGeM Classic kit (catalog no. 11-1250, Minerva Biolabs, Berlin, Germany).

### CAR123 production

#### Lentiviral particles production

The CAR lentiviral particles were produced in HEK293T cells as previously described.^13^ Briefly, HEK293T cells were co-transfected with pMDG (catalog no. 230420LNe-pMDG, RD-Biotech, Besançon, France), psPAX2 (catalog no. 230420LNe-psPAX2, RD-Biotech), and the plasmid carrying the cDNA encoding for the CAR in a medium enriched with calcium phosphate (Thermo Fisher Scientific, catalog no. J63122.AD). Two and 3 days later, the culture supernatant that contained the lentiviral particles was collected. The solution was then filtered and centrifuged overnight to concentrate the lentiviral particles. HEK293T cells were used for the titration of the concentrated supernatant.

#### CAR construct

A third-generation CAR-T cell targeting CD123 (CAR123) was generated using the previously described backbone and construction.^11^^,^^13^ The construct is composed of an EF1α promoter, an scFv targeting CD123, a CD28 transmembrane domain, CD28 and 4-1BB co-stimulatory domains (intracellular domain), a CD3ζ signaling domain, and a ΔCD19 (only extracellular/transmembrane domain) as a selection marker (Figure 1A).

#### CAR-T cells production

T cells used for CAR123 production were isolated from peripheral blood mononuclear cells (PBMCs) of healthy donors from the French Blood Transfusion Center (EFS, Besançon, France), all of whom consented to their blood being used for research purposes. PBMCs were extracted using lymphocyte separation medium gradient (catalog no. CMSMSL01-0U, Eurobio) and T cells were sorted and activated by CD3/CD28 activation beads (catalog no. 40203D, Gibco). The T cells were cultivated in RPMI medium supplemented with 8% human serum (EFS), 1% antibiotics, and 500 UI/mL IL-2 (PROLEUKIN, 18 million UI, AMM no. 5621586, Novartis, Basel, Switzerland). Two days after activation, T cells were transduced with a multiplicity of infection (MOI) of 10. Untransduced T cells (C0) were kept as a negative control. Seven days following transduction, the sorting and transduction efficiency was evaluated by flow cytometry with CD3-FITC and CD19-APC panels. For CAR123 production in the presence of dasatinib, 50 nM dasatinib was added 1 day after transduction. Beginning on day 3 post-transduction, cells were centrifuged and resuspended every 2 days at 1 × 10^6^ cells/mL in fresh medium with or without 50 nM dasatinib. Transduction efficiency was assessed on days 7 and 12 post-transduction by flow cytometry with CD3-BV421 and CD19-APC. CAR123 cells produced with dasatinib were sorted on day 7 using the same staining panel. One day prior to co-culture experiments, a subset of CAR123 produced in the presence of dasatinib was washed, centrifuged, and resuspended at 1 × 10^6^ cells/mL in fresh medium without dasatinib. CAR123 cells produced without dasatinib were extemporaneously treated or not with 50 nM dasatinib at the start of the co-culture assays as positive control of inhibition.

### Chemical reagents

Dasatinib (BMS-354825, CAS no. 302962-49-8) was purchased from Sigma-Aldrich (catalog no. SML2589) and reconstituted at a concentration of 2 mg/mL in dimethyl sulfoxide (DMSO) (catalog no. WAK-DMSO-70, WAK-Chemie Medical GmbH, Steinbach/Ts, Germany). Ponatinib (AP2453A, CAS no. 943319-70-8) was purchased from Selleckchem (Houston, TX, catalog no. S1490) and reconstituted at a concentration of 73 mg/mL in DMSO. Ruxolitinib (CAS no. 941678-49-5) was purchased from Selleckchem (catalog no. S1378) and reconstituted at a concentration of 61 mg/mL in DMSO.

### Flow cytometry

The cells were labeled and incubated with coupled antibodies, after which they were washed and analyzed by flow cytometry. The samples were analyzed using a BD FACS CANTO II, LSR Fortessa, or the BD FACSymphony A1 cytometer, and the data were processed using BD FACSDiva software version 9.0 (BD Biosciences, Franklin Lakes, NJ). The cytometry data from proliferation assays were analyzed with FlowJo software version 10.8.0 (Tree Star, Ashland, OR). To calculate the RFI, the mean fluorescence intensity of the studied condition was divided by that of the isotype control.

The materials and reagents used were as follows: from Miltenyi Biotec (Bergisch Gladbach, Germany), CD3-VioBlue (catalog no. 130-113-133; RRID: AB_10831672); from Sony Biotechnology (San Jose, CA), CD19-APC (catalog no. 2111060), CD123-PE/Cy7 (catalog no. 2130050), immunoglobulin G1 (IgG1) k-PE (phycoerythrin)/Cy7 (catalog no. 2600630), 7-AAD Viability Staining Solution (catalog no. 2702020), CD69-PerCP-Cy5.5 (catalog no. 2154630), IgG1 k-PerCP-Cy5.5 (catalog no. 2600750), CD25-FITC (fluorescein isothiocyanate) (catalog no. 2380530), IgG1 k-FITC (catalog no. 2600550), CD8-FITC (catalog no. 2323520), CD3-FITC (catalog no. 2101530), CD123-PE (catalog no. 2130030), hCD45-APC (allophycocyanin) (catalog no. 2120060), CD3-BV421 (catalog no. 2102170), CD19-PE/Cy7 (catalog no. 2111080), CD8-BV510 (catalog no. 2323660), CD45RA-PE/Cy7(catalog no. 2120630), CD45RO-PerCP-Cy5.5 (catalog no. 2121110), TNF-α-FITC (catalog no. 3114530), IFN-γ-BV785 (catalog no. 3112710), and IL-2-PerCP-Cy5.5 (catalog no. 3101610); from BioLegend (San Diego, CA), CD8-APC (catalog no. 344722); from BD Pharmingen, CD107a-PE (catalog no. 555801; RRID: AB_396091), IgG1 k-PE (catalog no. 555749; RRID: AB_396091), CD95-PE (catalog no. 555674; RRID: AB_396027); from Invitrogen (Waltham, MA), eBioscience Cell Proliferation Dye eFluor 450 (catalog no. 65-0842-85), eBioscience Cell Stimulation Cocktail (catalog no. 00-4970), and eBioscience Fixable Viability Dye eFluor 780 (FVD780) (catalog no. 65-0865-14); from BD Biosciences, GolgiStop (catalog no. 51-2092KZ) and GolgiPlug (catalog no. 555029); from R&D Systems (Minneapolis, MN), CCR7-FITC (catalog no. FAB197F) and IgG2A-FITC (catalog no. IC003F); and from Agilent Technologies (Santa Clara, CA), Dako IntraStain (catalog no. K231111-2).

For staining with eFluor 450 proliferation dye, T cells were resuspended in PBS 1× at a concentration of 0.5 × 10^6^ cells/mL and stained with a final concentration of 2.5 μM eFluor 450 proliferation dye, which was prediluted at 5 μM for killing, activation, and proliferation assays. In the degranulation assay, eFluor 450 was prediluted to 2.5 μM and used at a final concentration of 1.25 μM. Subsequently, the cells were incubated for 15 min at 37°C in the dark, with agitation every 5 min. The staining process was stopped by the addition of 10 mL of RPMI medium at 4°C supplemented with 10% stromal vascular fraction and 1% penicillin-streptomycin. The cells were incubated for 5 min at 4°C in the dark before centrifugation.

### CAR123 functionality

In all experiments, a co-culture of CAR123 or C0 with the target cell lines was conducted at an E:T ratio of 1:1 for a period of 18–24 h and with different doses of dasatinib and ponatinib (25–500 nM).

Prior to co-culture, effectors were labeled with eFluor 450 proliferation dye to allow for differentiation between effectors and target cells. In experiments comparing CAR123 manufacturing with or without dasatinib, eFluor 450 staining was replaced by staining with CD3-BV421 before analyzing by flow cytometry.

#### T cell activation assay

To evaluate the activation status of CAR123 before co-culture, CAR123 or C0 were labeled with eFluor 450. Briefly, CAR123 or C0 and CAL-1 or SKM-1 were co-cultured for 18 h with or without dasatinib or ponatinib (25–250 nM) in 96-well round-bottom plates (catalog no. 353077, Corning, Corning, NY). PMAi (cell stimulation cocktail) was used as positive control of activation. Cells were then washed, and membrane labeling was performed using CD19-APC, CD69-PerCP-Cy5.5, CD25-FITC, and PD-1-PE/Cy7 or isotypes before analyzing by flow cytometry to evaluate the expression level of activation markers on CAR123 (eFluor 450^+^/CD19^+^) or C0 (eFluor 450^+^/CD19^−^).

#### IFN-γ and TNF-α titration

To evaluate cytokine secretion (IFN-γ and TNF-α) of CAR123 or C0 in the presence or absence of dasatinib and ponatinib (25–250 nM), we carried out co-culture and titrated the supernatant. Co-culture was conducted between CAR123 or C0 and CAL-1 or SKM-1 in 24-well plates (catalog no. 353047, Corning) in 1 mL medium. After 24 h, the co-culture media were collected and stored at −80°C until titration. IFN-γ and TNF-α secretions were quantified by ELISA (catalog no. 851.560.005, Human IFN gamma ELISA Set, Diaclone, Besançon, France; catalog no. 851.570.005, Human TNF alpha ELISA Set, Diaclone; catalog no. 858.000.005, Accessory Pack for ELISA Sets, Diaclone) according to the manufacturer’s protocol.

### T cell degranulation assay

To evaluate T cell degranulation, we carried out co-culture of CAR123 or C0 with CAL-1 or SKM-1 in 96-well round-bottom plates, with and without the addition of dasatinib or ponatinib (25–250 nM). PMAi was used as a positive control of degranulation. Anti-CD107a-PE or isotype was added at the start of the co-culture. After 1 h of incubation, GolgiStop was added to each well. After a total of 5 h of incubation, cells were marked with 7-AAD, CD19-APC, and CD8-FITC before analyzing by flow cytometry.

#### Proliferation assay

To evaluate the effect of dasatinib on the proliferation of CAR123 of C0, we carried out co-culture with the CAL-1 cell line. Before co-culture, CAR123 or C0 were dyed with eFluor 450 proliferation dye. Co-cultures were done with CAR123 or C0 and CAL-1 in 96-well round-bottom plates with an E:T ratio of 1:1 with or without dasatinib (25–250 nM). CAR123 and C0 alone treated with 250 nM ruxolitinib were used as a negative control. Cells were incubated for 3 days before staining with CD3-FITC and CD19-APC before analyzing by flow cytometry. The division index, defined as the mean number of divisions undergone by each cell from the initial generation, was calculated for CD3^+^/CD19^+^ (CAR123) and CD3^+^/CD19^−^ (C0) cells using FlowJo software. The position of the undivided cell peak, which is essential for the calculation, was determined using ruxolitinib control. Ruxolitinib is a highly specific JAK1/2 inhibitor, and it is known to inhibit T cell proliferation by blocking cytokine receptor signaling. CAR123 and C0 labeled by antibodies but not stained with eFluor 450 were used for the evaluation of background fluorescence, which is also necessary for the calculation.

#### T cell cytotoxicity assay

To evaluate CAR123-mediated cytotoxicity, CAR123 or C0 were labeled with eFluor 450. After 24 h of co-culture between CAR123 or C0 and CAL-1 or SKM-1 in the E:T ratio of 1:1 with or without dasatinib and ponatinib (25–250 nM) in a 96-well round-bottom plate, cells were stained with 7-aminoactinomycin D (7-AAD) and analyzed by flow cytometry to evaluate target cell death. To evaluate the reversibility of dasatinib and ponatinib inhibitory effects, CAR123 was treated with or without dasatinib and ponatinib (100–500 nM) for 24 h. Following treatment, the cells were washed and co-cultured for 24 h with CAL-1 or SKM-1 in a 96-well round-bottom plate. Untreated CAR123 were treated with dasatinib and ponatinib (100–500 nM) at the start of the coculture as a control of inhibition. After co-culture, cells were stained with 7-AAD and target cell death was evaluated by flow cytometry.

#### Intracellular cytokine evaluation

To assess cytokine production (IFN-γ, TNF-α, and IL-2) by CAR123 produced in the presence or absence of dasatinib (50 nM), CAR123 were co-cultured with CAL-1 in 96-well round-bottom plates, with and without the addition of dasatinib (50 nM). PMAi was used as positive control for cytokine production. After 1 h of incubation, GolgiPlug was added to each well. Following a total incubation time of 6 h, cells were stained with FVD780, CD19-APC, CD3-BV421, and CD8-BV510. Intracellular staining then was performed using TNF-α-FITC, IFN-γ-BV785, and IL-2-PerCP-Cy5.5 using Dako IntraStain according to the manufacturer’s protocol before analyzing by flow cytometry.

#### Memory phenotype evaluation

The differentiation profile of CAR123 produced with or without dasatinib was evaluated on day 12 post-transduction. T cells were stained with CD3-BV421, CD8-BV510, CD19-APC, CD45RA-PE/Cy7, CD45RO-PerCP-Cy5.5, CCR7-FITC, and CD95-PE. Analysis by flow cytometry was performed on the CD19^+^ population to determine the frequency of naive (CD45RA^+^/CD95^−^/CCR7^+^), stem cell-like memory (CD45RA^+^/CD95^+^/CCR7^+^), central memory (CD45RO^+^/CD95^+^/CCR7^+^), effector memory (CD45RO^+^/CD95^+^/CCR7^+^) and effector (CD45RA^+^/CD95^+^/CCR7^−^) T cell subsets.

#### Transwell model and CD123 expression by HMEC-1 and HUVEC

To assess the effect of proinflammatory cytokines on CD123 expression in endothelial cells, we used transwell co- or tri-culture model. For this purpose, HMEC-1 cells or HUVEC were seeded in a 24-well plate. A thincert cell culture insert (0.4 μM pore size, catalog no. 662640, Greiner Bio One, Kremsmünster, Austria) was added to each well. Dasatinib was added in the media, and a co-culture with CAR123 or C0 and CAL-1 was then carried out in the upper chamber. After 24 h, HMEC-1 or HUVEC were digested by trypsin and marked with CD123-PE/Cy7 or isotype to evaluate CD123 expression by flow cytometry. As a control, HMEC-1 were cultured alone with or without dasatinib and without thincert culture insert in HMEC-1 culture media. CD123 expression was evaluated as described before.

#### Real-time cytotoxicity assay

To evaluate CAR123 toxicity on HMEC-1 and CAL-1 in a pro-inflammatory environment, we co-cultured HMEC-1 or CAL-1 with CAR123 in conditioned media in an Incucyte S3 (Incucyte S3 Live Cell Analysis Instrument, Sartorius, Göttingen, Germany). Co-culture was done in 24-well plates with CAR123 and CAL-1 with an E:T ratio of 1:1 with or without dasatinib. After 24 h of co-culture, conditioned media was collected and centrifuged to discard cells. HMEC-1 or CAL-1 (as a positive control) were washed, resuspended in PBS, and stained with 0.75 μg/mL of CellTracker Deep Red Dye (Cat# C34565, Thermo Fisher Scientific). Following a 20-min incubation at 37°C in the dark, staining was stopped with culture medium, and cells were incubated for 5 min at 4°C in the dark. Each well of a 96-well flat-bottom plate (catalog no. 353072, Corning) was seeded with HMEC-1 or CAL-1. HMEC-1 were incubated for 24 h in the incubator in HMEC-1 culture media and washed before the assay to remove unadhered cells. CAL-1 were seeded just before the assay. In co-culture conditions CAR123 or C0 were added at an E:T ratio of 1:1. Co-cultures were made in conditioned media with or without dasatinib treatments (25, 50, 100 and 250 nM). TNF-α and IFN-γ blocking antibodies (catalog nos. SIM0001 and BE0235, BioXCell, Lebanon, PA) were both used at 5 μg/mL. Incucyte Cytotox Green Dye (catalog no. 4633, Sartorius) was added in each well with a final concentration of 1× to stain dead cells. Four scans were conducted per well, with a magnification of 10, a phase analysis, and acquisitions of 300 and 400 ms for the red and green channels, respectively. These were performed at 2-h intervals over a 48-h period. The mortality rate of the target cells (calculated as the ratio of the simultaneously red and green cell number to the total red cell number) was determined using the Incucyte 2023A Rev2 software. The target cell mortality rate in co-culture was normalized with the target-only condition to obtain the cytotoxicity of CAR123 or C0.

#### *In vitro* modeling of CAR-T cells functionality with limited duration dasatinib treatment

To assess the effect of a 50-nM dasatinib treatment of a limited duration on CAR123 IFN-γ and TNF-α secretion and cytotoxicity on CAL-1, a co-culture of CAR123 or C0 with CAL-1 at an E:T ratio of 1:1 was conducted with or without 50 nM dasatinib. After a 3-h, 6-h, or 9-h incubation period, the treated cells were centrifuged. The medium was then collected for cytokine titration, and the cells were reseeded with fresh media without dasatinib. Following a total co-culture period of 24 h, untreated cells and cells that have been treated for 3, 6, 9, or 24 h were centrifuged. The medium was collected for IFN-γ and TNF-α titration as previously described. To evaluate target cell death, cells were stained with CD3-BV421 and 7-AAD and analyzed by flow cytometry.

#### Recursive killing assay with *in vitro* dasatinib treatment cycle

To evaluate the effect of dasatinib in a re-stimulation situation. A model was created to simulate *in vitro* the effect of dasatinib treatment cycles on CAR123 cytotoxicity against CAL-1. We carried out the co-culture of CAR123 or C0 with CAL-1 at varying or stable concentrations of dasatinib or vehicle. CAR123, C0, and CAL-1 were seeded at an E:T ratio of 1:5 in a 24-well plate in a final volume of 1 mL.

In the treatment cycle conditions, on day 0, cells were treated with 50 nM dasatinib for 3 h. Then, half the medium was replaced with fresh medium to halve the dasatinib concentration to 25 nM. Nine hours later, dasatinib was added to the medium to reach a final concentration of 50 nM. This 12-h treatment cycle was repeated 9 times, after which the cells were centrifuged and the medium was completely replaced with fresh medium without dasatinib.

In the stable 50 nM dasatinib conditions, cells were continuously treated with 50 nM dasatinib. Medium was replaced at the same time as the treatment cycle conditions, but cells were retreated to maintain 50 nM dasatinib.

In the vehicle condition, cells were treated with vehicle. Medium was replaced at the same time as the treatment cycle conditions, but cells were re-treated with vehicle to maintain vehicle concentration equivalent to 50 nM dasatinib.

After 48 and 96 h of co-culture, 200 μL of co-culture was collected after 2 to 3 h of 50 nM dasatinib treatment to assess the absolute number of T cell and CAL-1 in each well by using Trucount Absolute Counting Tubes IVD (catalog no. 663028, BD Biosciences). Cells were marked with 7-AAD, CD3-VioBlue, CD123-PE, and hCD45-APC before analyzing by flow cytometry. We removed 300 μL from each well, and the CAR123 and CAL-1 co-culture wells were supplemented with fresh CAL-1 to restore the E:T ratio to 1:5. Then, we added 500 μL fresh medium to all wells to reach 1 mL.

#### Jurkat TPR CAR123 production and assay

Jurkat TPR were transduced with an MOI of 10. Twelve days post-transduction, CD19^+^ cells were sorted by flow cytometry. To assess activation and inhibition of NF-κB, AP-1, and NFAT trancriptional activity, we carried out co-culture with CAL-1 or SKM-1 cell lines. After 24 h of co-culture between Jurkat TPR CAR123 and CAL-1 or SKM-1 in an E:T ratio of 1:1 with or without dasatinib (25–250 nM) in a 96-well round-bottom plate, cells were stained with FVD780, CD8-APC, and CD19-PE/Cy7 and analyzed by flow cytometry to evaluate expression of EGFP, CFP, or mCherry, which are produced upon activation of NFAT, NF-κB, and AP-1 transcriptional activity, respectively. PMAi was used as a positive control for activation.

### Statistical analysis

All data are presented as mean ± standard deviation (SD) except Figures 3A–3H, S5G, and S5H, which are shown as median ± quartile, and Figures 6E, 6F, and S3G, which are shown as mean ± standard error of the mean. The data were compared with a one-way ANOVA followed by Tukey’s multiple comparison test. The real-time cytotoxicity data were compared with a two-way ANOVA, followed by Tukey’s multiple comparison test. All statistical analyses were performed using GraphPad Prism 9.0.0 (GraphPad Software, La Jolla, CA). The statistical significance is indicated in the figures according to the *p* value code: ∗*p* < 0.0332, ∗∗*p* < 0.0021, ∗∗∗*p* < 0.0002, and ∗∗∗∗*p* < 0.0001.

## Data and code availability

The data that support the findings of this study are included in the paper or may be available from the corresponding author.

## Acknowledgments

We thank the Agence Nationale de la Recherche (ANR) under the program “Investissements d’Avenir” (ANR-11-LABX-0021-LipSTIC). This study was supported by the Association Laurette Fugain (ALF 2021/09), Programme de Recherche Translationnelle INCa (PRT-K-20-107), Bionoveo, and the Région Bourgogne-Franche-Comté. We thank Dr. Sylvain Simon (Fred Hutchinson Cancer Research Center, Seattle, WA), for providing the Jurkat 76 TPR cells.

## Author contributions

F.G.-O. and M.F. designed the project. F.G.-O., F.R., and M.F. supervised the research. C.-F.M., M.F., A.L., and T.L. performed the experiments. C.-F.M. and M.F. analyzed the data. S.B., M.F., X.R., G.R., B.R., E. D., O.A., R.L., and E.B.-R. provided guidance and expertise in their respective areas of study. C.-F.M., M.F., and F.G.-O. wrote the manuscript. All authors contributed to the manuscript and approved the final version.

## Declaration of interests

F.G.-O., E.B.-R., and O.A. have filed for intellectual patent rights on aspects of the current research (WO2020254682A1) licensed to CARLA Biotherapeutics. F.G.-O. and O.A. are cofounders of CARLA Biotherapeutics. M.F. is an employee of CARLA Biotherapeutics. CARLA Biotherapeutics did not provide any financial support for this work.

## Supplemental information

Document S1. Figures S1–S6

Document S2. Article plus supplemental information
