# Supplementary material for: Evaluation of dasatinib and ponatinib for the control of CD123 CAR-T cell functionalities
Source: Mol Ther Oncol. 2025 Nov 21;33(4):201097. doi: 10.1016/j.omton.2025.201097 (PMC12752794; doi:10.1016/j.omton.2025.201097)
Supplement: Document S1. Figures S1–S6 [file mmc1.pdf]

OMTON, Volume *33*

Supplemental information

Evaluation of dasatinib and ponatinib for the control of CD123 CAR-T cell functionalities

Charles-Frédéric Mantion, Sabeha Biichlé, Xavier Roussel, Gwenaël Rolin, Agathe Lejeune, Tony Labaigt, Elodie Bôle-Richard, Etienne Daguindau, Bernard Royer, Florian Renosi, Olivier Adotevi, Romain Loyon, Maxime Fredon, and Francine Garnache-Ottou

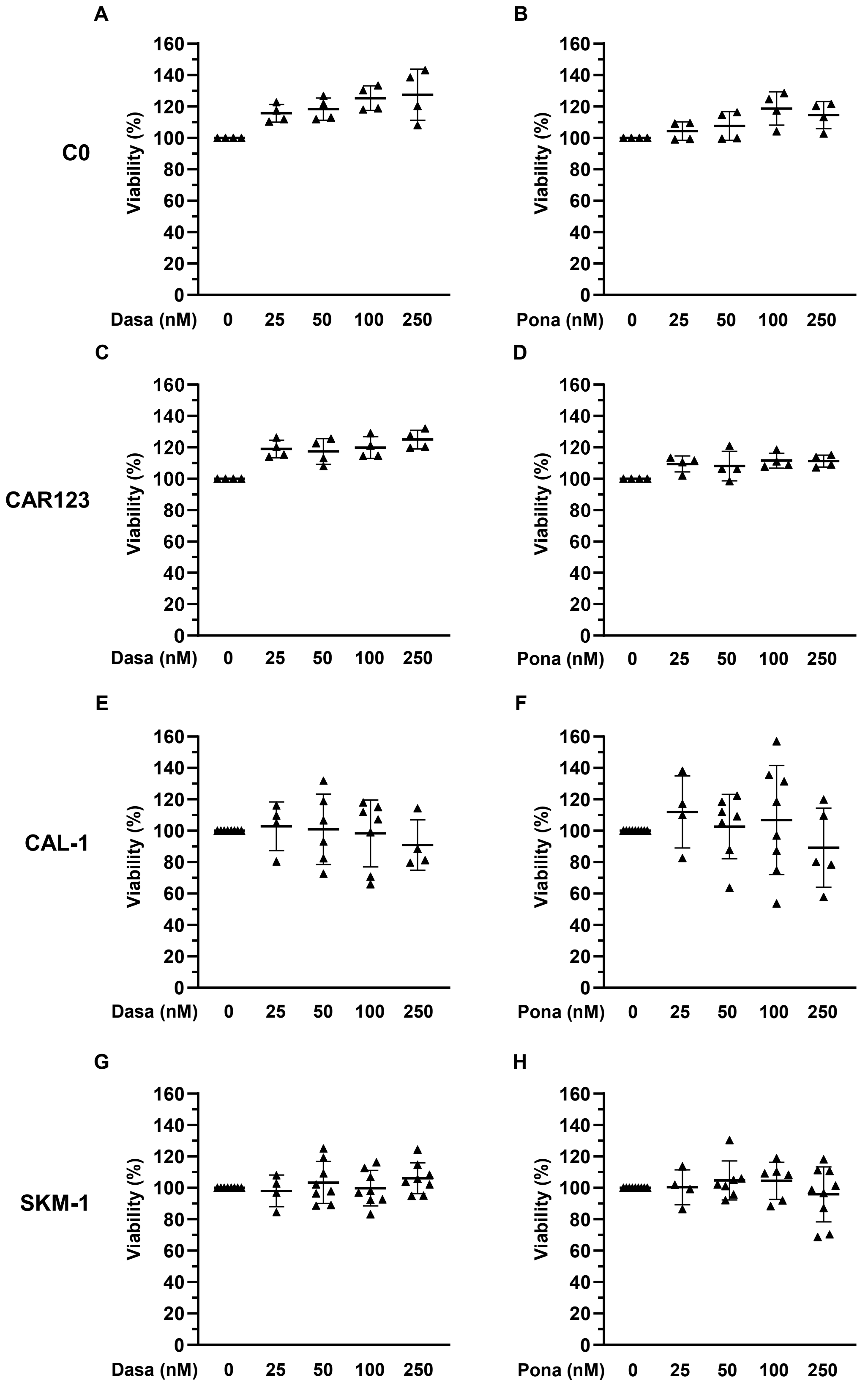

Figure S1. Effect of dasatinib and ponatinib on cell viability
C0 (**A and B**), CAR123 (**C and D**), CAL-1 (**E and F**) or SKM-1 (**G and H**) were cultured for 24 hours with or without dasatinib (**A, C, E and G**) (25 to 250 nM) or ponatinib (**B, D, F and H**) (25 to 250 nM). Viability of cells was assessed by 7-AAD labeling in flow cytometry and normalized by viability of untreated cell (n ≥ 4 in all experiments). Data shown as mean ± SD.

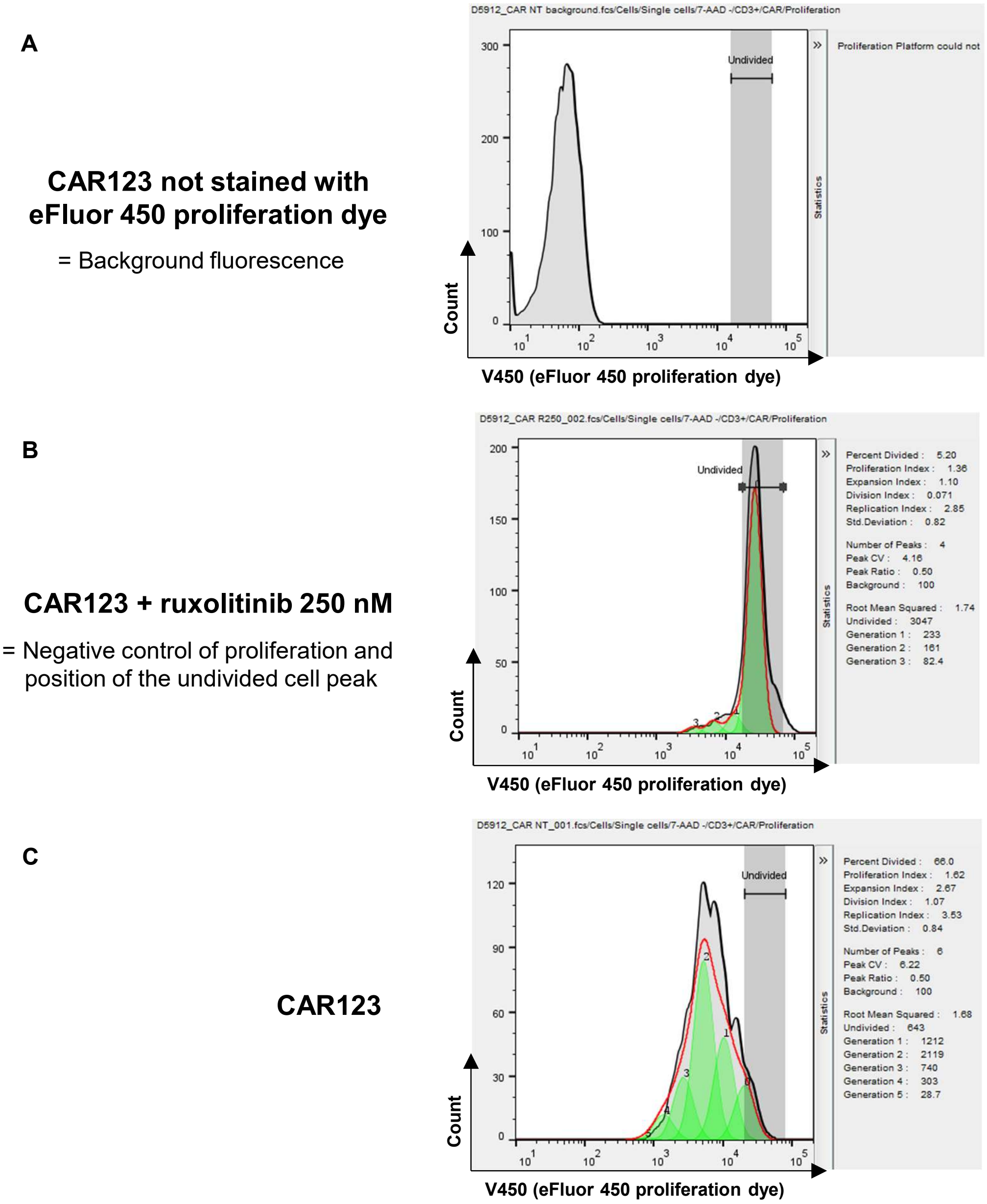

Figure S2. Proliferation analysis with FlowJo software
FlowJo models the position of the peak of each generation in a sample and provides proliferation-related statistic. The difference between the proliferation model and the empirical data is represented by the root mean squared value. A number of variables can be manipulated in order to align the model as closely as possible to the sample and to minimize the root mean squared: Position of the undivided peak, background fluorescence, coefficient of variation of peaks (peak width, called peak CV), variation of fluorescence between each peak (peak ratio) and the number of peaks to be modeled. The sum of events in all peaks is visualized with a red line. (**A**) CAR123 not stained with the proliferation dye, used to define background fluorescence. (**B**) Negative control for proliferation, used to define the position of undivided cells. Ruxolitinib inhibits T cell proliferation when cultured alone. (**C**) Example of sample analyzed: untreated CAR123 cultured alone.

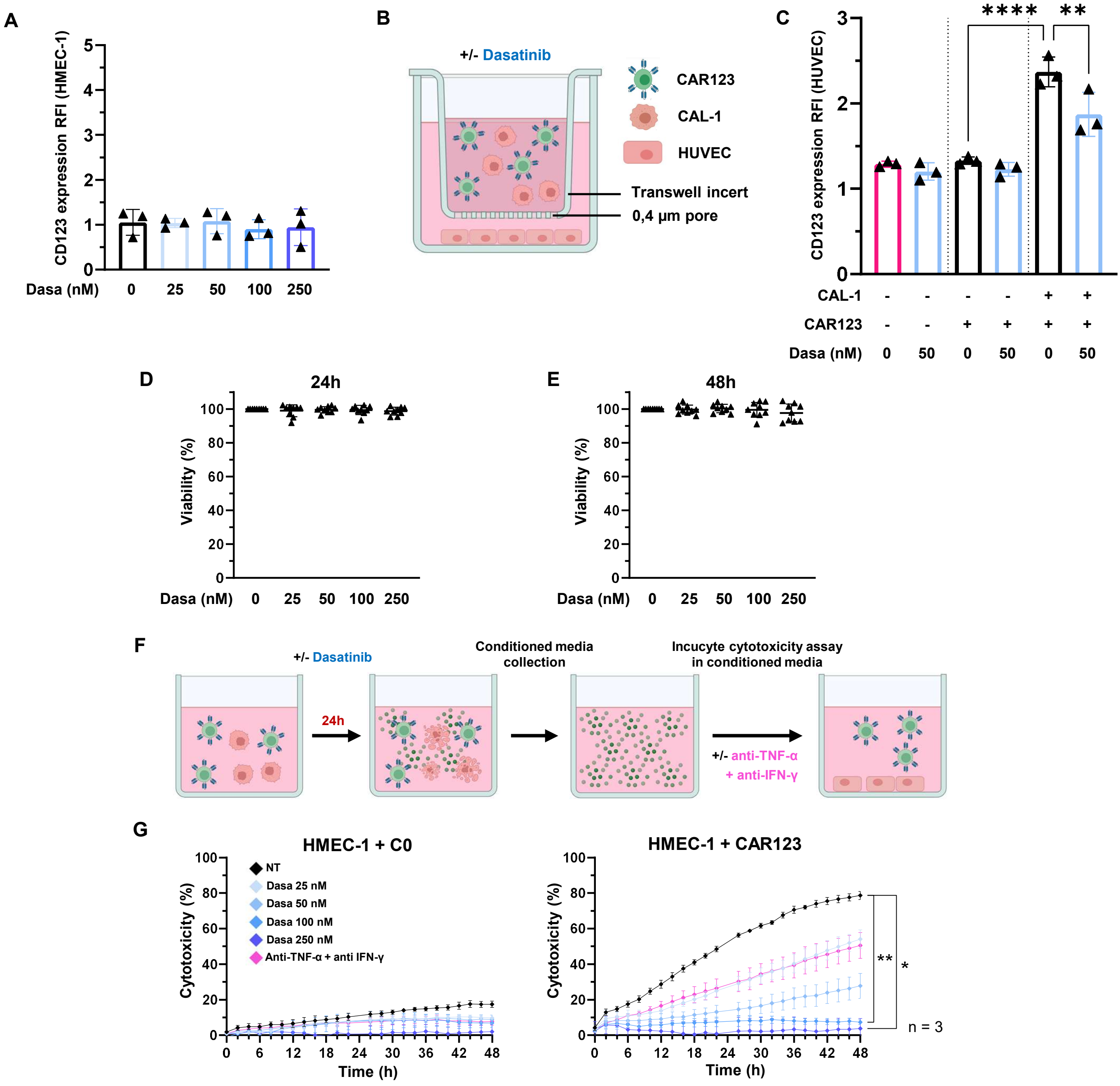

Figure S3. Modulation of the on-target/off-tumor effect of CAR123 by dasatinib

(**A**) CD123 RFI of HMEC-1 after 24 hours culture with dasatinib (25 to 250 nM). Data shown as mean ± SD. $p < 0.0332$ (*), $p < 0.0021$ (**), $p < 0.0002$ (***), $p < 0.0001$ (****) determined with ordinary one-way ANOVA with Tukey's multiple comparison test. Values compared to untreated HMEC-1. (**B**) Schematic of the Transwell co-culture model. CAR123 were co-cultured with CAL-1 at E:T ratio = 1:1 in the upper chamber with or without dasatinib, HUVEC were seeded in the lower chamber. Following 24 hours of culture HUVEC were digested with trypsin to harvest a single cell suspension and CD123 RFI was evaluated by flow cytometry. (**C**) CD123 RFI of HUVEC in the previously described model (n = 3 T cell donors, n = 1 HUVEC donor). Data shown as mean ± SD. $p < 0.0332$ (*), $p < 0.0021$ (**), $p < 0.0002$ (***), $p < 0.0001$ (****) determined with ordinary one-way ANOVA with Tukey's multiple comparison test. Viability of HMEC-1 after 24 hours (**D**) and 48 hours (**E**) of treatment with dasatinib (n = 8). HMEC-1 cell death was followed by the Incucyte S3 system and normalized by untreated HMEC-1 cell viability. Data shown as mean ± SD. (**F**) Schematic of the Incucyte S3 model with conditioned media production. CAR123 was co-cultured with CAL-1 for 24 hours with or without dasatinib (25 to 250 nM) before collection of the conditioned supernatant. (**G**) HMEC-1 were co-cultured for 48 hours at E:T ratio = 1:1 with C0 (left) or CAR123 (right) in conditioned T cell media containing dasatinib (25 to 250 nM) or TNF-α/IFN-γ blocking antibodies (5 µg/mL of each) (n = 3 donors). HMEC-1 cell death was followed by the Incucyte S3 system and normalized with viability of target cultured alone. Data shown as mean ± SEM. Ordinary two-way ANOVA with Tukey's multiple comparison test. $p < 0.0332$ (*), $p < 0.0021$ (**), $p < 0.0002$ (***), $p < 0.0001$ (****). Values compared to untreated CAR123.

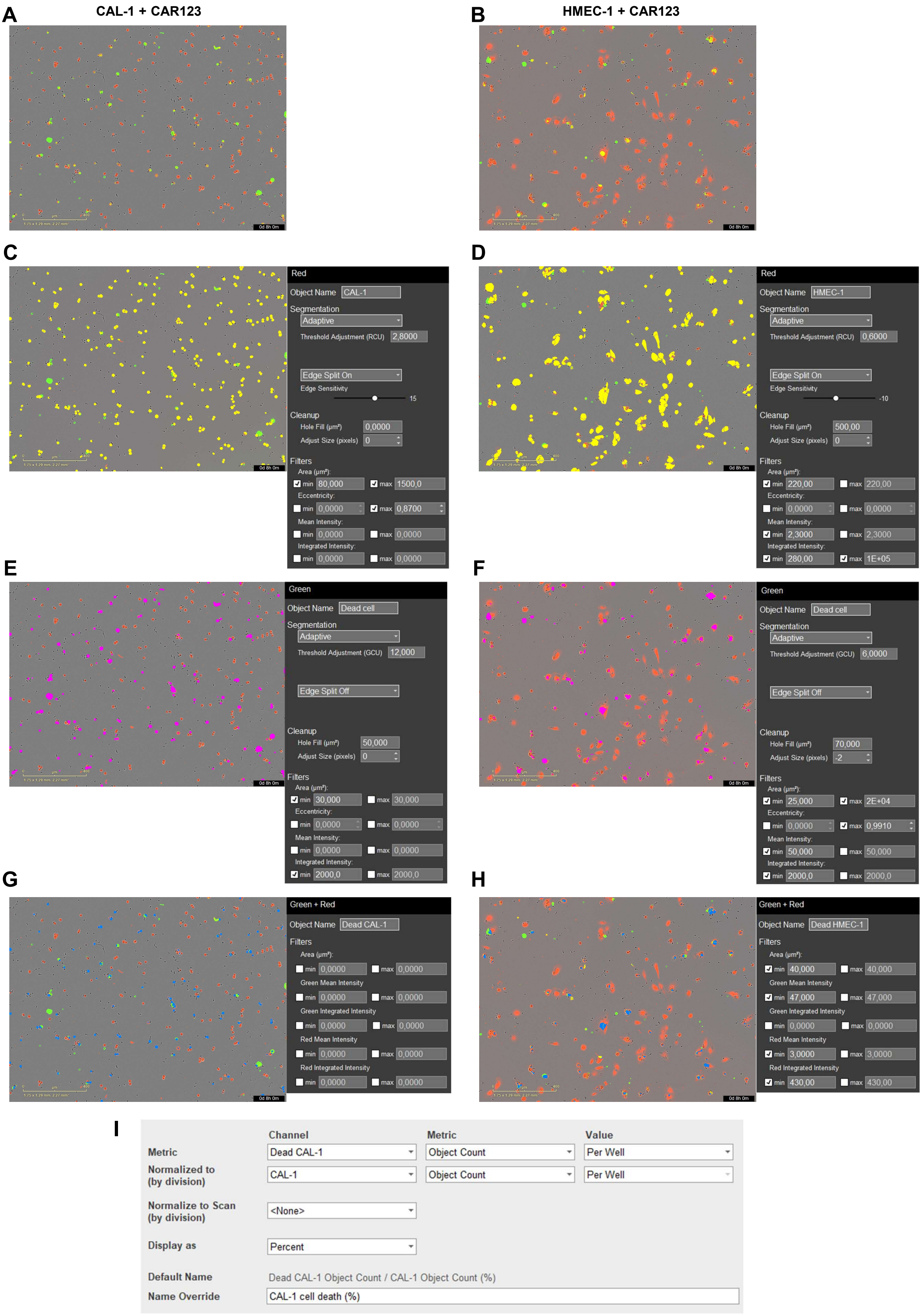

J

$$\textbf{Cytotoxicity}\ (\%) = (\text{target cell death \% in coculture} - \text{target cell death \% when alone}) \times \frac{100}{100 - \text{target cell death \% when alone}}$$

Figure S4. Cell viability analysis with Incucyte 2023A Rev2 software

(**A, C, E and G**) Analyzed area containing CAR123 and CAL-1 after 8 hours of co-culture. (**B, D, F and H**) Pictures and screenshot of the analysis of a quart of a well containing CAR123 and HMEC-1 after 8 hours of co-culture. (**A and B**) Raw data consist of a phase-contrast photo, red and green fluorescence levels (**C and D**) Red fluorescence analysis: Red fluorescence superior to the background was included in the analysis and then fragmented so that one red event corresponds to one target cell object (yellow in the pictures). Events too small to be a cell are filtered. (**E and F**) Green fluorescence analysis: Green fluorescence that is high enough above the background is included in the analysis but not fragmented. It gives position of dead cells (pink in the pictures). Events too small or not fluorescent enough to be a dead cell are filtered. (**G and H**) Overlap analysis: Overlap of red and green analysis masks give position of dead targets cell object (blue in the pictures). One object corresponds to one dead target cell thanks to the fragmentation performed in the red fluorescence analysis. For HMEC-1, events corresponding to cellular debris that sedimented on top of live HMEC-1 are filtered based on size and red fluorescence parameters. (**I**) New unit definition screen. Target cell death % is generated for each well with the number of dead target cell object divided by the total number of target cell object. (**J**) Formula used to calculate C0 or CAR123 cytotoxicity on the target cell. C0 or CAR123-induced cell (CAL-1 or HMEC-1) death (referred as cytotoxicity) is calculated by using target cell death % when target cell are cultured alone and when they are co-cultured with C0 or CAR123 with the same concentration of treatment.

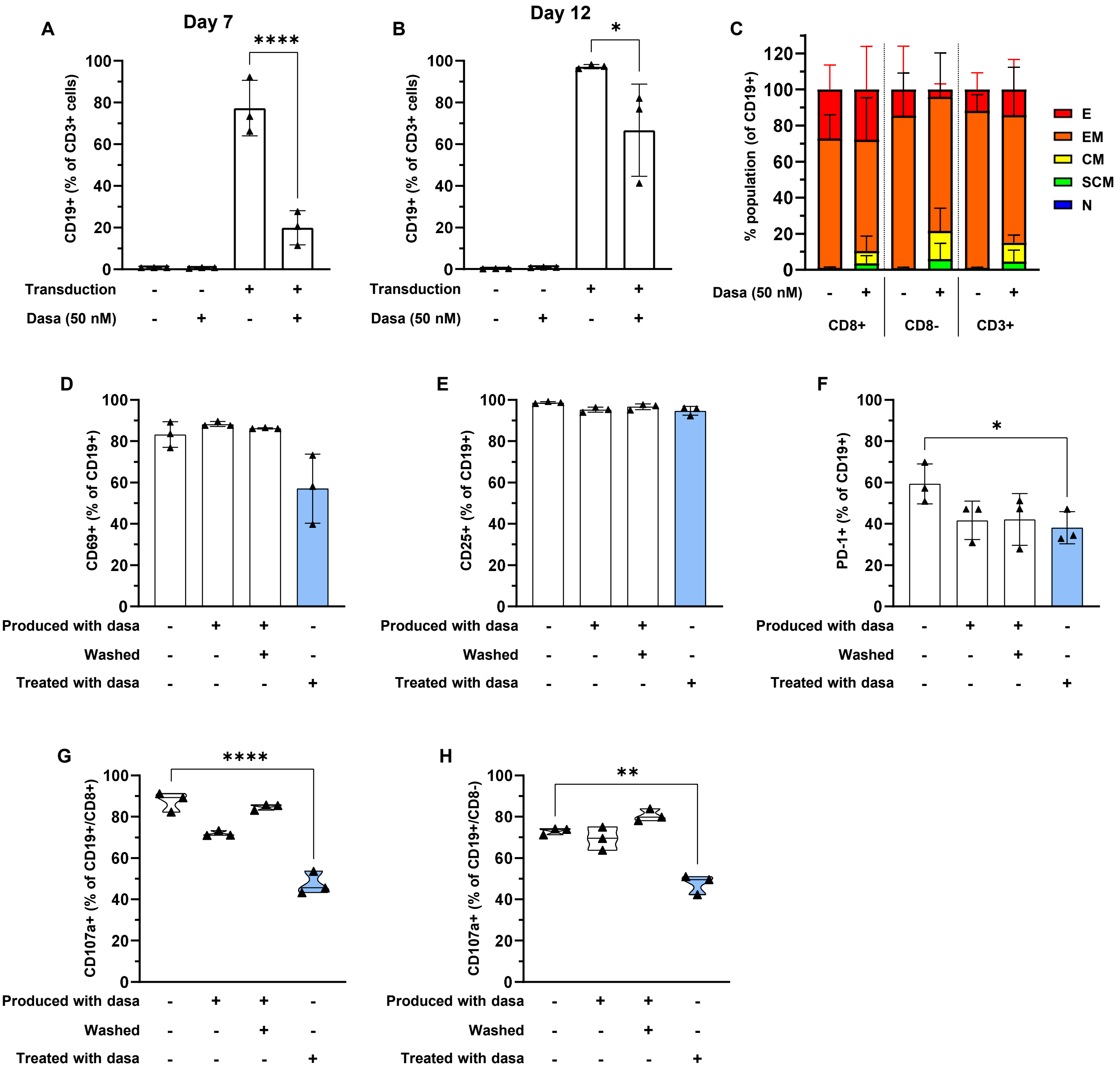

Figure S5. Production of CAR123 with dasatinib and modulation of CAR123 phenotype and functionality
CAR123 transduction efficiency on day 7 (**A**) and day 12 (**B**) post-transduction after production with or without dasatinib 50 nM (n = 3 donors). Data shown as mean ± SD. p < 0.0332 (*), p < 0.0021 (**), p < 0.0002 (***), p < 0.0001 (****) determined with ordinary one-way ANOVA with Tukey's multiple comparison test. Values compared between transduced T cell treated or not (**C**) Memory phenotype of CD8+, CD8- or all CAR123 on day 12 after production with or without dasatinib 50 nM (E: effector; EM: effector memory; CM: central memory; SCM; stem cell-like memory; N: naïve) (n = 3 donors). Data shown as mean ± SD. CAL-1 was co-cultured for 18 hours at E:T ratio = 1:1 with CAR123 produced with or without 50 nM dasatinib and washed or not 24 hours before experiment or CAR123 produced without dasatinib and extemporaneously treated with 50 nM dasatinib. CD69 (**D**), CD25 (**E**) and PD-1 (**F**) expression were evaluated by flow cytometry (n = 3 donors). CAL-1 was co-cultured for 5 hours at E:T ratio = 1:1 with CAR123 produced with or without 50 nM dasatinib and washed or not 24 hours before experiment or CAR123 produced without dasatinib and extemporaneously treated with 50 nM dasatinib. CD107a expression among CD8+ (**G**) and CD8- (**H**) CAR123 was evaluated by flow cytometry (n = 3 donors). Data shown as median ± quartile. p < 0.0332 (*), p < 0.0021 (**), p < 0.0002 (***), p < 0.0001 (****) determined with ordinary one-way ANOVA with Tukey's multiple comparison test. Values compared to untreated CAR123 in co-culture with CAL-1.

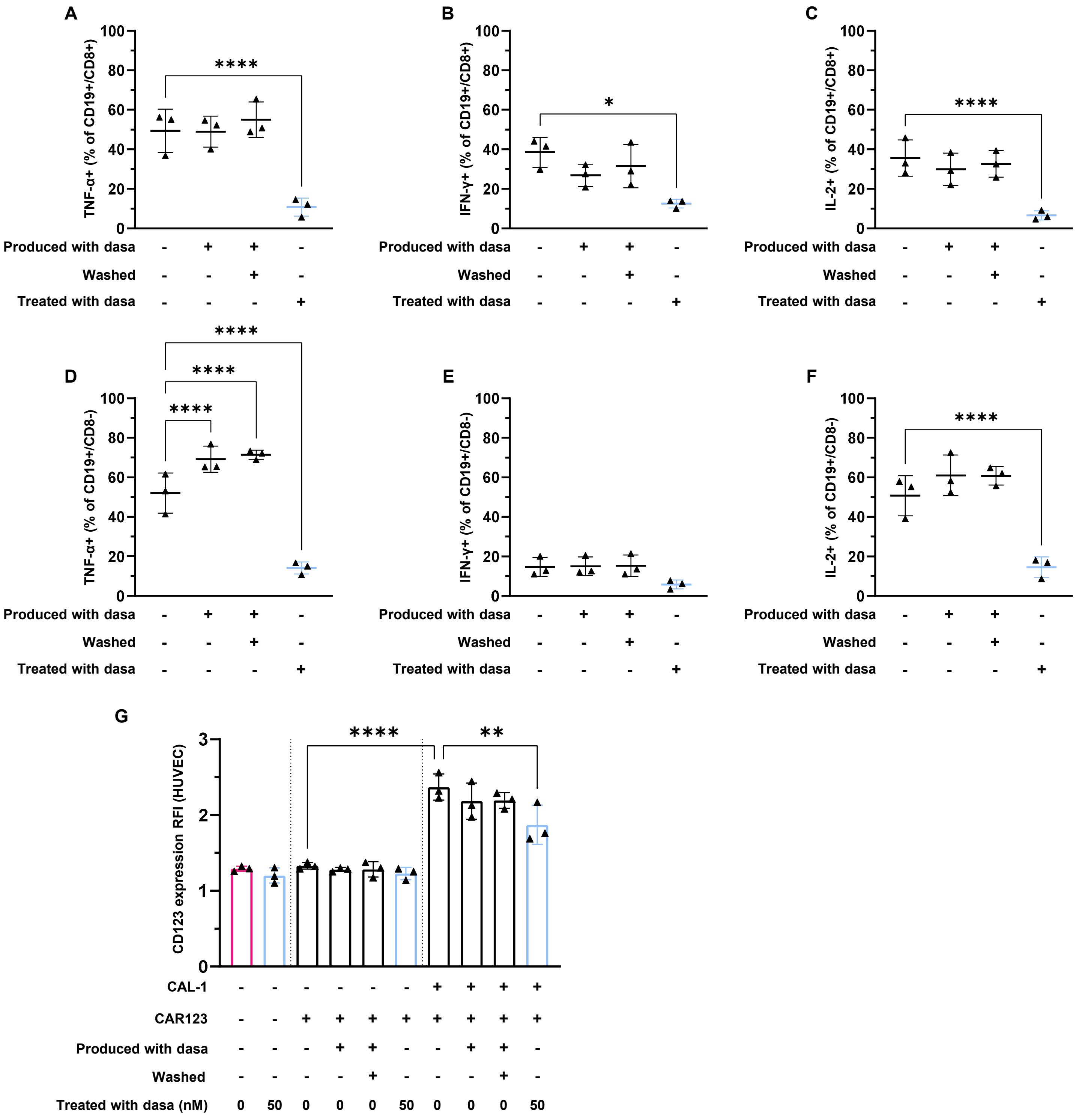

Figure S6. Production of CAR123 with dasatinib and modulation of CAR123 cytokine production
CAL-1 was co-cultured for 5 hours at E:T ratio = 1:1 with CAR123 produced with or without 50 nM dasatinib and washed or not 24 hours before experiment or CAR123 produced without dasatinib and extemporaneously treated with 50 nM dasatinib. TNF-α, (**A and D**) IFN-γ (**B and E**) and IL-2 (**C and F**) intracellular expression among CD8+ (**A, B and C**) and CD8- (**D, E and F**) CAR123 was evaluated by flow cytometry (n = 3 donors). Data shown as mean ± SD. $p < 0.0332$ (*), $p < 0.0021$ (**), $p < 0.0002$ (***), $p < 0.0001$ (****) determined with ordinary one-way ANOVA with Tukey's multiple comparison test. Values compared to untreated CAR123 in co-culture with CAL-1. (**G**) CAR123 produced with or without 50 nM dasatinib and washed or not 24 hours before experiment were co-cultured with CAL-1 at E:T ratio = 1:1 in the upper chamber and extemporaneously treated or not with dasatinib (50 nM), HUVEC were seeded in the lower chamber. Following 24 hours of culture HUVEC were digested with trypsin to harvest a single cell suspension and CD123 RFI was evaluated by flow cytometry (n = 3 T cell donors). Data shown as mean ± SD. $p < 0.0332$ (*), $p < 0.0021$ (**), $p < 0.0002$ (***), $p < 0.0001$ (****) determined with ordinary one-way ANOVA with Tukey's multiple comparison test. Values compared to untreated CAR123 in co-culture with CAL-1.

Supplemental videos captions:

Video S1. Representative Incucyte recording of CAR123 co-cultured with CAL-1

CAL-1 appear red. Dead cells appear green. Related to Figure 6E, right.

Video S2. Representative Incucyte recording of CAR123 co-cultured with CAL-1 with 50 nM dasatinib

CAL-1 appear red. Dead cells appear green. Related to Figure 6E, right.

Video S3. Representative Incucyte recording of CAR123 co-cultured with HMEC-1

HMEC-1 appear red. Dead cells appear green. Related to Figure 6F right.

Video S4. Representative Incucyte recording of CAR123 co-cultured with HMEC-1 with 50 nM dasatinib

HMEC-1 appear red. Dead cells appear green. Related to Figure 6F, right.
